# Recombination, admixture and genome instability shape the genomic landscape of *Saccharomyces cerevisiae* derived from spontaneous grape ferments

**DOI:** 10.1371/journal.pgen.1011223

**Published:** 2024-03-22

**Authors:** Chris M. Ward, Cristobal A. Onetto, Steven Van Den Heuvel, Kathleen M. Cuijvers, Laura J. Hale, Anthony R. Borneman

**Affiliations:** 1 Australian Wine Research Institute, Urrbrae, South Australia, Australia; 2 University of Adelaide, Adelaide, South Australia, Australia; Faculdade de Ciências e Tecnologia, Universidade Nova de Lisboa, PORTUGAL

## Abstract

Cultural exchange of fermentation techniques has driven the spread of *Saccharomyces cerevisiae* across the globe, establishing natural populations in many countries. Despite this, Oceania is thought to lack native populations of *S*. *cerevisiae*, only being introduced after colonisation. Here we investigate the genomic landscape of 411 *S*. *cerevisiae* isolated from spontaneous grape fermentations in Australia across multiple locations, years, and grape cultivars. Spontaneous fermentations contained highly recombined mosaic strains that exhibited high levels of genome instability. Assigning genomic windows to putative ancestral origin revealed that few closely related starter lineages have come to dominate the genetic landscape, contributing most of the genetic variation. Fine-scale phylogenetic analysis of loci not observed in strains of commercial wine origin identified widespread admixture with European derived beer yeast along with three independent admixture events from potentially endemic Oceanic lineages that was associated with genome instability. Finally, we investigated Australian ecological niches for basal isolates, identifying phylogenetically distinct *S*. *cerevisiae* of non-European, non-domesticated origin associated with admixture loci. Our results illustrate the effect commercial use of microbes may have on local microorganism genetic diversity and demonstrates the presence of non-domesticated, potentially endemic lineages of *S*. *cerevisiae* in Australian niches that are actively admixing.

## Author summary

*Saccharomyces cerevisiae* yeast are utilized throughout the world to carry out fermentation and have been transferred between cultures for thousands of years. However, Australia is thought to lack natural populations of this yeast. In this study we investigated the genomic landscape of *S*. *cerevisiae* isolated from spontaneous wine fermentations in Australia, identifying widespread admixture and genome instability. Local phylogenetics identified clear signals for admixture between basal non-wine isolates, some of which were found to be associated with previously unidentified yeast from Australian natural environments. This discovery suggests that natural non-domesticated lineages of *S*. *cerevisiae* exist in the Australian environment that may predate colonisation. Finally, we investigated genetic contribution against a publicly available set of commercial starter and wine strains isolated in Australia over the past 70 years. Revealing that few related commercial starter lineages contribute most of the genetics within the standing variation of the spontaneously fermenting yeast analysed in this study.

## Introduction

The yeast *Saccharomyces cerevisiae* has been tied to human culture and movement since Neolithic communities in the Middle- and Far- East began fermenting fruits and grain 8000–9000 years ago [1,2]. Phylogenomic evidence further supports this, with natural isolates from forests of modern China and South East Asia forming the crown node to all domesticated isolates of *S*. *cerevisiae* [3,4]. Grapes and their associated fermentation products are then thought to have spread through trade and dispersion into the Near East and East Mediterranean regions [5], resulting in the endemic wild populations identified in China (CN) [3]; Taiwan (TW) [6]; Japan [7]; Europe [7]; North America (NA) [7]; Africa [8] and South East Asia [8] forming clear taxonomic clades [3,4,6,8].

Historically, wine was fermented spontaneously, with inoculation by autochthonous (native/endemic/feral/wild) yeast occurring through environmental transfer [9–11]. Potentially inoculating yeast, that derive from domesticated or wild lineages present in the environment, into the ferment. This had the potential to confer unique and terroir-dependent sensory traits to wine that would otherwise be absent [12–14]. In contrast, most modern industrial-scale fermentations are inoculated using commercial starter cultures containing a yeast monoculture [9,15,16], providing predictability of fermentation characteristics [12,17]. However, introduction of monoculture into the environment at high frequency has been shown to affect the population structure of local microbes [18]. The frequency and quantity of commercial inoculations may therefore have significant effects on the genomic landscape and strain biodiversity in regions thought to be devoid of native strains, such as in Australia. Furthermore, starter culture transferred into the environment may undergo feralization through admixture with other domesticated and/or non-domesticated lineages present within the environment.

Admixture and horizontal gene transfer has been essential in driving the diversification of *S*. *cerevisiae* [19–21], with admixture from basal Asian isolates into wine populations being proposed as likely the origin of the Beer2 clade that is associated with modern ale production [19]. Widespread use, and subsequent dispersal, of domesticated strains has been a major contributor to the frequency of admixture [22] and has led to the establishment of diverse feral populations throughout the world. Feral *S*. *cerevisiae* have been isolated in low frequencies from the guts of insects, grape vines, and fermentation related machinery, providing multiple distinct avenues for isolates to be inoculated into the fermentation medium from environmental reservoirs [7,21,23,24]. Despite this, key questions regarding the genetic composition of *S*. *cerevisiae* populations remain.

Oceania is thought to lack native *S*. *cerevisiae* populations, with the presence of this yeast only being described after wine and beer production began in the late 19^th^ century [25]. In contrast, evidence exists for Indigenous Australian’s carrying out fermentation utilizing non-*Saccharomyces* yeast lineages [26]. In this study, whole-genome resequencing was performed on 411 *S*. *cerevisiae* strains that were isolated from spontaneous fermentations from grape varieties across a five-year period in Australian wineries. Through comparison against 169 wine isolates and 91 diverse yeast isolates [3,7,8], the genomic landscape of spontaneous wine yeast shows evidence of complex recombination between commercial starter haplotypes coupled with admixture events from beer and basal lineages suggesting the majority of yeast found in these spontaneous wine fermentations had undergone feralization. After screening cultures of Australian wild niches, isolates were identified that were separate from known phylogenetic clades, providing evidence that non-domesticated, potentially endemic lineages exist in Australia.

## Results

### Isolate origin and genotyping

Extracts from spontaneous fermentations from five grape varieties (Viognier, Pinot Noir, Shiraz, Grenache, Chardonnay and Cabernet Sauvignon) were cultured and isolates with ITS sequences matching *Saccharomyces cerevisiae* (n = 434) were sequenced using short read Illumina platforms outputting an estimated mean coverage of 29.9x (95% CI: 28.5x-31.3x) per isolate. Short reads were investigated for species origin using exact 31-mer matches against the genomes of *sensu stricto Saccharomyces* and out of 434 sequenced spontaneous isolates, 427 were classified as *S*. *cerevisiae* (S1 Fig). 31-mers originating from a non-*S*. *cerevisiae* origin, ie. through inter-species introgression or incorrect ITS assignment, would be assigned to their species of origin, providing genomic position agnostic species assignment. No significant signal for between species hybridisation was identified, with >99% of classified samples k-mers matching *S*. *cerevisiae*. After filtering for average read depth (DP≥6) and genotyped coverage (>80% of S288C genome [27]), a total of 411 spontaneous isolates, along with 169 reference yeast genomes [16] and 91 diverse [3,7,8] publicly available yeast isolates were retained for analysis (S2 Fig and S1 Table). The reference panel utilized contains publicly available genomes from many of the available commercial starter cultures utilized in Australia (n = 102) along with non-starter yeast isolated over the past 70 years [16].

### Recent admixture between spontaneous *S*. *cerevisiae* and non-domesticated isolates

**Fig 1:**
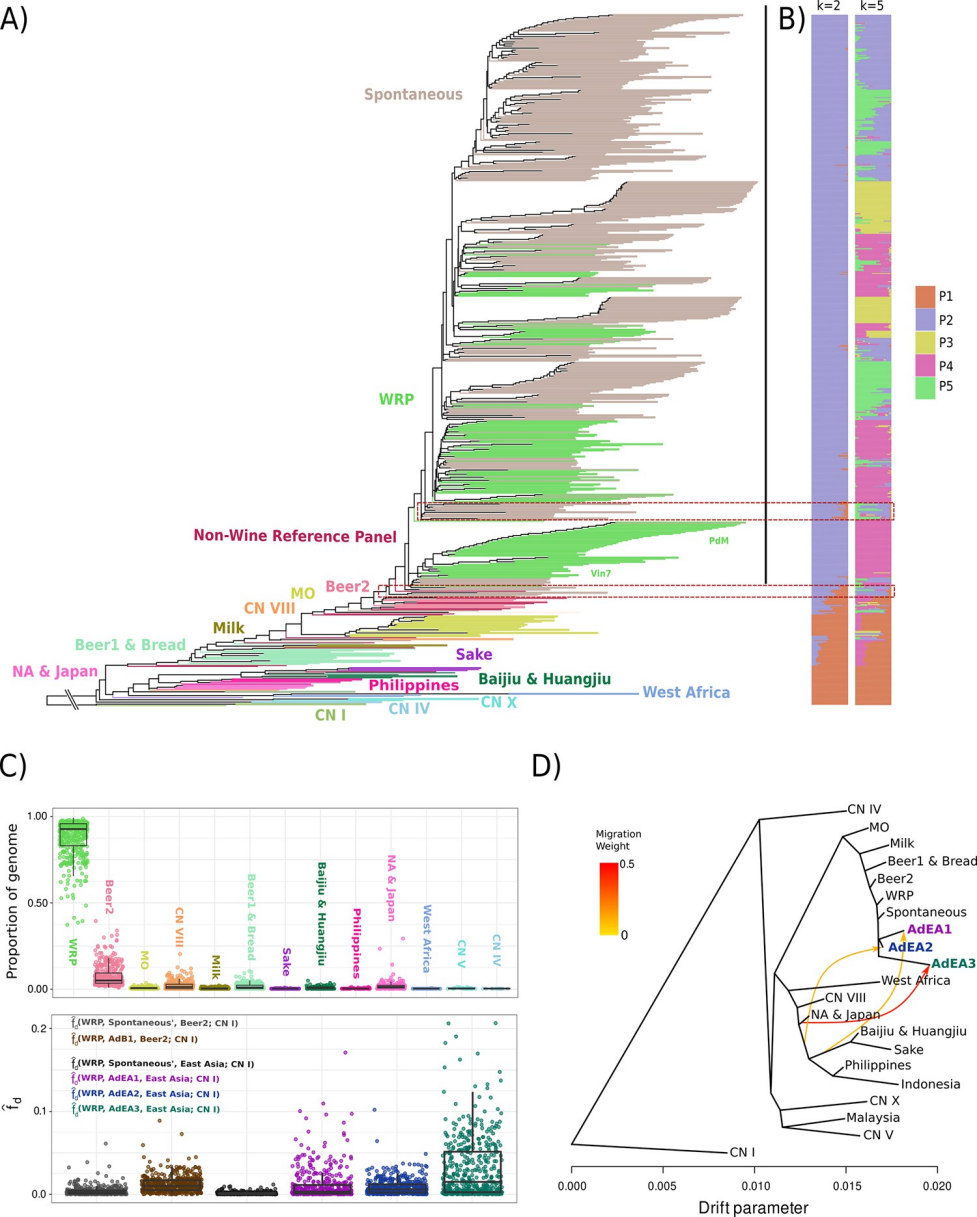
Ancestry inference of spontaneous isolates identified three distinct admixture events from basal Asian clades. A) Whole genome species tree reconstructed from 1386 single copy ortholog gene trees colored according to a priori populations. Black bar represents the bounds of the European Wine cade and defines the Wine Reference Panel (WRP). B) Genome wide estimation of drift histories for k = 2 & 5. C) Upper: The proportion each spontaneous isolates genome that was most closely related to one of the clades in A using 10kb genomic windows of Nei’s da. Lower: $\hat{f_{d}}$ tests calculated across 25kb windows to identify allelic bias from Beer2 or North America/East Asia (a combined population of Baijiu & Huangjiu, CN VIII and North America (NA) & Japan isolates) present in admixed pseudo-populations that is absent from WRP isolates. $\hat{f_{d}}$ replacing admixed pseudo-populations with Spontaneous* isolates (Spontaneous isolates are not in AdB1, AdEA1, AdEA2 or AdEA3) used as a negative control. Boxes showing the first and third quartile range (IQR) while whiskers extend to a maximum of 1.5 * IQR. D) Drift tree with putative admixed individuals treated as separate populations (AdEA1, AdEA2, AdEA3) allowing for directional ‘migration’ events to simulate admixture between populations. Simplified version of S5 Fig m = 10. All populations are colored based on clade assignment in panel A.

To investigate the genetic provenance of the spontaneous isolates, phylogenetic reconstruction was carried out utilizing the full set of 411 spontaneous and 169 reference isolates, in addition to a publicly available set of 91 diverse yeast from major domesticated and non-domesticated clades described in Pontes, Hutzler (4). The resultant phylogeny matched previous studies with disparate brewing methods separating into well supported clades (Fig 1A). Reference panel isolates were mostly monophyletic, bifurcating into the three previously described clades Vin7, PdM and a mixed clade of other European wine representatives [16] with isolates of non-wine origin being assigned to their respective clades (eg. strain 931 sake). Isolates within the reference set that were placed within the wine clade (Fig 1A; black bar) were defined as the Wine Reference Panel (WRP) and contained both commercial wine starter (n = 102) and non-starter wine clade isolates (n = 82).

Nine spontaneous isolates were in paraphyly with WRP wine samples suggesting admixture with other ancestral isolates may have occurred. ADMIXTURE analysis with *a priori* population number (K) set from 2–5 was carried out to investigate the drift ancestry of each of the isolates, clearly separating samples into European derived, bounded by the Mediterranean Oak (MO) MRCA, and basal ancestries (Figs 1B and S3). Mixed ancestries were identified for the “Beer2”, “MO” and “Beer1 & Bread” clades, in agreement with previous work [6,7,19,20]. Paraphyletic and spontaneous isolates at the base of the major European wine clade showed a clear mixture of multiple ancestries at all values of K, reinforcing the prediction that these are the result of mixed descent (Fig 1B; red boxes).

Pairwise Nei’s ancestral distance (*d*_*a*_) [28] was then calculated across 25kb tiled windows between individual spontaneous isolates and all members of each clade to determine if any spontaneous loci were genetically closer to non-wine isolates than WRP isolates (Fig 1C). *d*_*a*_ estimates the genetic distance between ancestral nodes by removing within clade nucleotide diversity, making it a more accurate estimator of strain origin than conventional genetic distance. The proportion of windows most similar to WRP isolates ranged from 0.37–0.99 of the total identifiable genetic contribution for each spontaneous isolate (Fig 1C). Non-WRP windows were most associated with the Beer2 clade, contributing between 0.009 and 0.40 of the windows in any strain (Fig 1C). Significant proportions of windows derived from the clades “China (CN) VIII”, “North America & Japan”, “Huangjiu & Baijiu” and “Beer1 & Bread” were also observed (Fig 1C). Spontaneous wine isolates with genomic contribution from non-wine lineages were compared to phylogenetic (Fig 1A and 1B) and PCA (S4 Fig) outliers to resolve four pseudo-populations; one population containing 58 samples with mixed Wine-Beer2 ancestry (AdB1) and three candidate admixed populations (AdEA1, AdEA2 and AdEA3), which contained windows with the closest *d*_*a*_ to modern North American and East Asian lineages (Fig 1C). Of these admixed isolates, the AdEA1 population was the most common containing 33 samples isolated at a single location (W5) in two different grape variety ferments (Shiraz, Chardonnay) across 3 years (2016–2018). Population AdEA2 contained individuals (n = 5) from two locations (W4, W5) sampled in 2016 and 2018, whereas AdEA3 contained individuals (n = 2) from a single location (W5) and year (2018).

Window-wise admixture population allele frequency was then tested for allelic bias from the Beer2 or North American/East Asian (Baijiu & Huangjiu, China (CN) VIII and North America (NA) & Japan isolates) populations that was absent from WRP and all non-admixed spontaneous isolates (Spontaneous’) using the $\hat{f_{d}}$ statistic [29] (Fig 1C). The $\hat{f_{d}}$ statistic utilizes shifts in derived/ancestral biallele frequency within a population to estimate if the biallele has been inherited in a tree-like fashion, or if ancestral variance has been inherited in the test population that is absent from the neighbouring branch. Control tests were used to determine the baseline $\hat{f_{d}}$ value that is expected under the species tree when admixed isolates are removed. Control tests $\hat{f_{d}}$ (WRP, Spontaneous’, Beer2; CN I) (95% CI: 0.0032–0.004; max: 0.069) and $\hat{f_{d}}$ (WRP, Spontaneous’, East Asia, CN I) (95% CI: 0.0012–0.0014; max: 0.012) showed little deviation from the expected ~0 value under a topology absent of admixture. However, tests using admixed populations displayed increased $\hat{f_{d}}$ (Fig 1C): $\hat{f_{d}}$ (WRP, AdB1, Beer2; CN I) (95% CI: 0.0114–0.0130, max: 0.089), $\hat{f_{d}}$ (WRP, AdEA1, East Asia; CN I) (95% CI: 0.009–0.012; max: 0.171), $\hat{f_{d}}$ (WRP, AdEA2, East Asia; CN I) (95% CI: 0.008–0.01; max: 0.1) and $\hat{f_{d}}$ (WRP, AdEA3, East Asia; CN I) (95% CI: 0.027–0.033, max: 0.21) suggesting loci have introgressed from basal lineages phylogenetically associated with the East Asian/North American lineages, but dissimilar from those previously sequenced.

A drift tree was then constructed with the admixed populations separated from the bulk of the spontaneous isolates, reconstructing the demographic history of *S*. *cerevisiae* strains while accounting for between-clade gene flow (Fig 1D). Iterating through migration edge values from 6 through 10 (S5 Fig) revealed widespread admixture between clades, likely contributing to the phylogenetic inconsistency reported in previous work [3,4,6]. After removing known admixture events [4,19,20], the three novel admixture events were confirmed as originating from unknown source lineages most related to modern East Asian or North American populations (Fig 1D). The source lineage for the AdEA1 admixture event was estimated to have diverged from a common ancestor of East Asian domesticated ferments and contributed 0.12 drift to the AdEA1 population (Fig 1D). AdEA2 and AdEA3 sources were derived from likely non-domesticated lineages related to, but not a part of, the North America & Japan clade; contributing 0.14 and 0.44 drift respectively (Fig 1D).

### Multiple admixed haplotypes of divergent origin are segregating in spontaneous populations

**Fig 2:**
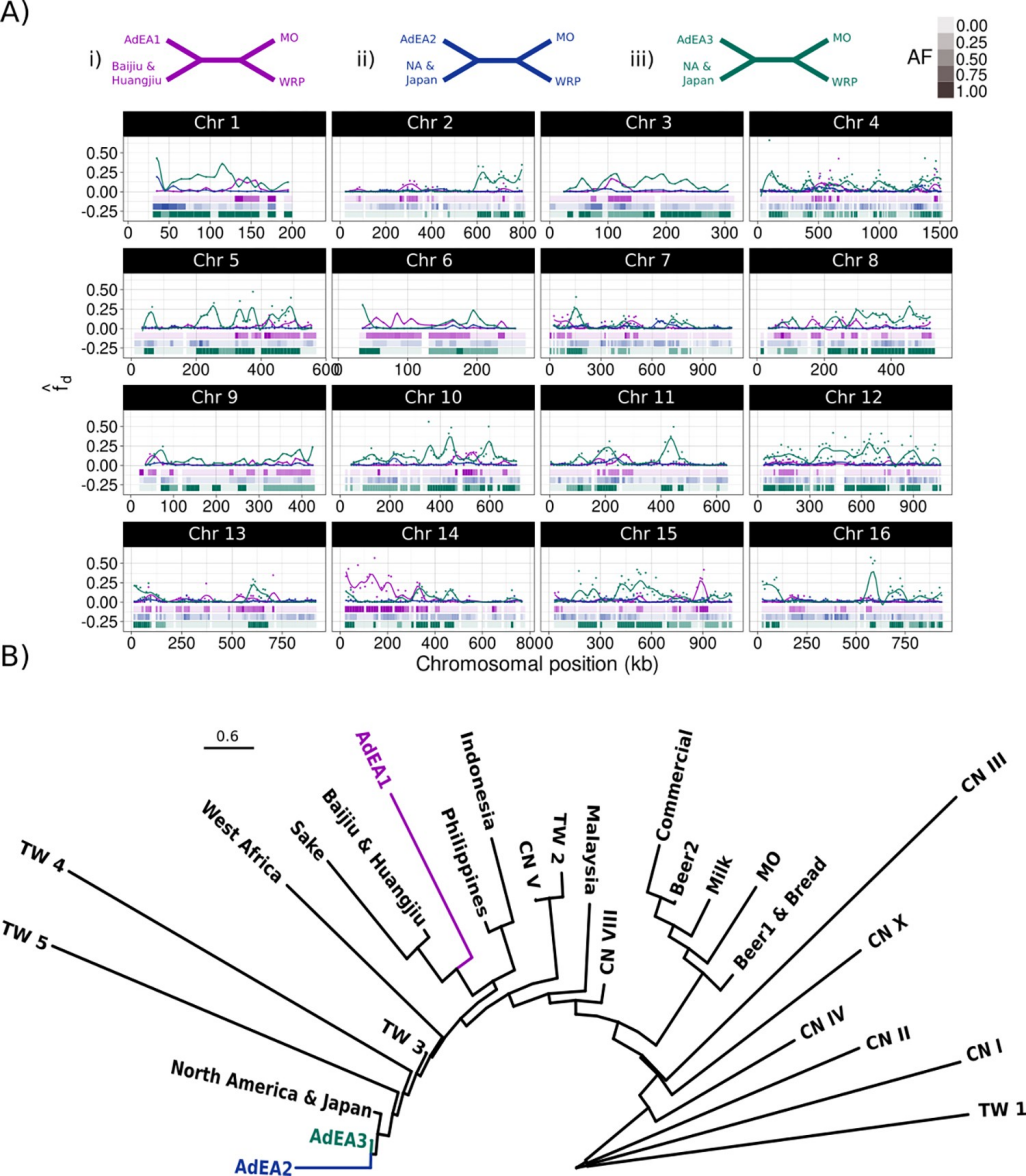
A) Genome wide windowed $\hat{f_{d}}$ and local topology reconstruction identified fixed admixture loci in populations AdEA1 (purple), AdEA2 (blue) and AdEA3 (green). Each $\hat{f_{d}}$ population was tested for their respective $\hat{f_{d}}$ and subtree (i-iii) at each 10kb tiled window across the genome: Admixed East Asia 1 $\hat{f_{d}}$ (WRP, AdEA1; Baijiu&Huangjiu, CN I), ii) Admixed East Asia 2 $\hat{f_{d}}$ (WRP, AdEA2; NA&Japan, CN I) and iii) Admixed East Asian 3 $\hat{f_{d}}$ (WRP, AdEA3; NA&Japan, CN I). Presence of admixture subtrees is shown as blocks below y = 0 for each of the tested subtrees (i-iii) with transparency indicating allele frequency (AF) in the population. Completely colorless windows are due to the local trees being unresolved. B) Coalescent species tree reconstruction of fixed, consecutive admixture loci for AdEA1, AdEA2 and AdEA3 against a background of diverse lineages rooted using CN I, TW 1 and CN II populations. As no loci were fixed in AdEA2 a single homozygous diploid isolate from pop AdEA2 was used (Q-3_S92). Scale is in coalescent units. An expanded tree with posterior probabilities can be found in S7 Fig.

To confirm the presence of North American/East Asian admixture and identify the breakpoints of admixed loci, two complementary approaches were applied to 10kb windows of computationally phased genotypes: $\hat{f_{d}}$ and local phylogenetic reconstruction. Signals of phylogenetic incongruence in these approaches were considered signals for admixture, confirming the presence of three distinct admixture events into spontaneous isolates (Fig 2A and S2 Table). As the admixture source population is unsampled for each of the putative admixture events, local 10kb phylogenies were broken down into subtrees containing the test spontaneous ferment isolate, the closest sampled lineage to the putative admixture source, MO isolates and WRP wine isolates. Subtrees were considered incongruent if the spontaneous isolate shared a most recent common ancestor (MRCA) with the closest sampled lineage (eg North America & Japan) while not in monophyly with MO and WRP isolates (Fig 2A i-iii).

**Fig 3:**
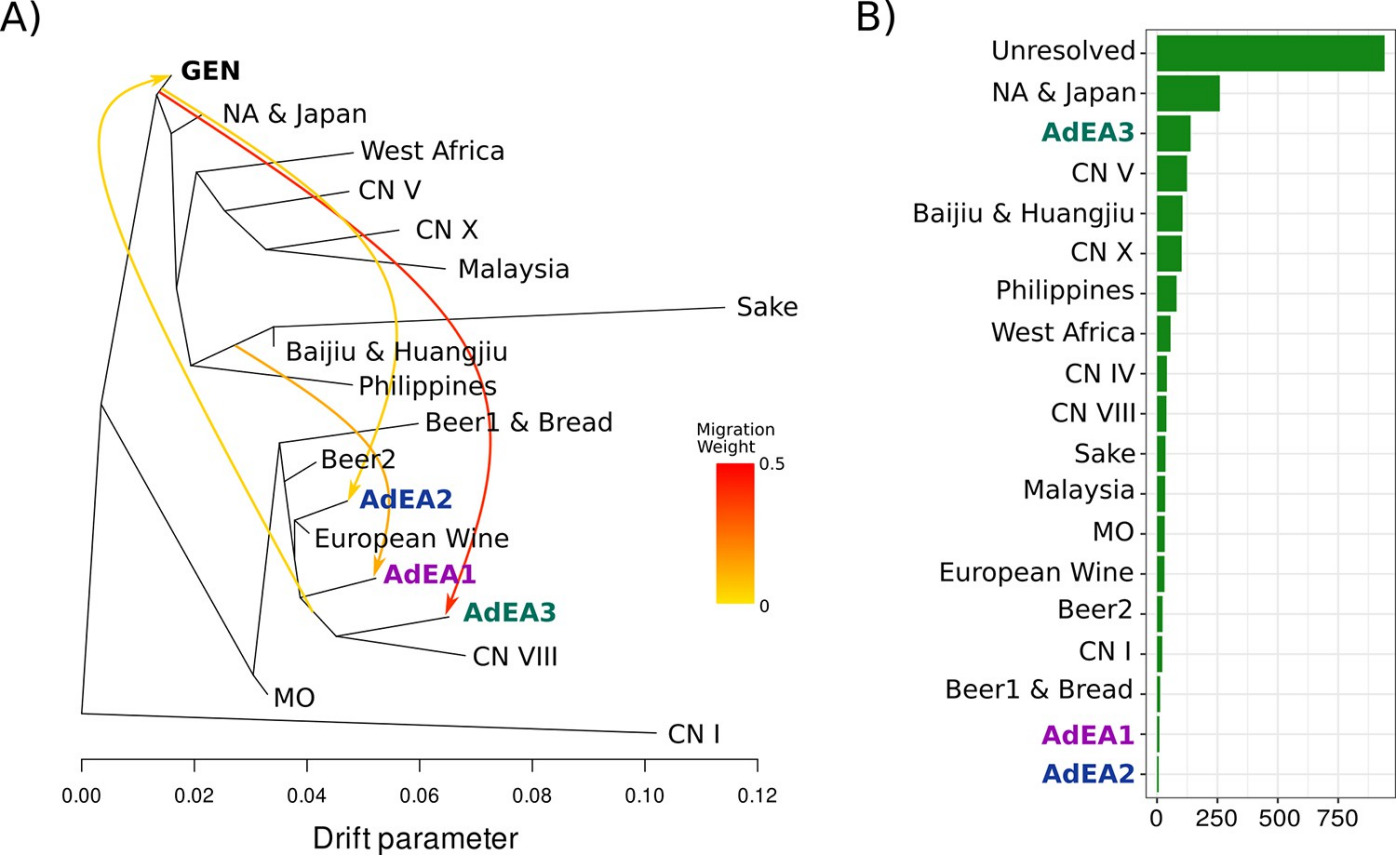
Evolutionary history of Saccharomyces cerevisiae isolated from Australin ecological niches. A) Drift tree reconstructed from segregating biallele frequency within 911 single copy ortholog alignments generated from genes predicted within genome assemblies of Australian natural isolates (GEN), diverse yeast isolates, and Admixed spontaneous isolates. B) The number of gene trees with GEN samples in reciprocal monophyly with one of the clades in A. Gene trees where GEN is monophyletic or closes neighbours with two or more different clades are labelled as “Unresolved”.

Population AdEA1 showed clear signs of admixture from an unknown isolate originating before the MRCA of Sake and Baijiu & Huangjiu across 2.71 Mb (22.6% of genome) and fixed across 0.36 Mb (Fig 2A i). Due to the origin of the AdEA2 gene-flow event in the drift tree (Fig 1D) phylogenetic placement was not well resolved, yet using the North America & Japan clade as the source population coupled with local phylogenetics clearly highlighted genetic breakpoints of the gene-flow event across 5.92 Mb (49% of the genome), although none were fixed (Fig 2A ii). Population AdEA3 exhibited the largest total introgressed loci totalling 7.1 Mb (59% of genome) of which 3.7 Mb was fixed (Fig 3A iii). Furthermore, the highly recombined and heterozygous nature of many of the introgressed loci from the AdEA1 and AdEA2 populations (Fig 3A) suggests that the introgressed content is actively being removed from the standing variation. Yet some loci remained consistently fixed, for example, fixed loci in AdEA1 appeared stable over the 2016–2018 period in which they were observed (S6 Fig), while other loci changed allele frequency between years (S6 Fig). Phylogenetic reconstruction of homozygous admixture loci against a background of diverse populations, including recently identified Taiwanese wild populations [6] along with further Chinese [3] and South East Asian [8] isolates confirmed that they were distinct in respect to these known *S*. *cerevisiae* lineages (Figs 2B and S7) and derive near estimated migration edges in the drift tree (Fig 1D). Population AdEA1 was derived from an unknown source lineage basal to East Asian domesticated yeast with high posterior support (Fig and S7). Topology further revealed that AdEA2, AdEA3, and North America & Japan may be derived from a common ancestor, though support for these nodes was lower (Fig 2B and S7).

### Non-European derived populations exist in Australian wild niches

The presence of non-domesticated ancestries segregating within *S*. *cerevisiae* isolated from spontaneous ferments suggests that multiple non-domesticated lineages are likely present within Australian natural yeast populations. To complement the analyses carried out on spontaneous wine isolates and determine a putative donor lineage, long read genome assemblies were produced for two strains (GEN12d2 and GEN58f3) that were originally isolated from environmental samples (plant and feather sample) collected from Bray Park, Queensland, Australia. GEN strains were chosen for sequencing due to their suspected non-European and non-domesticated ancestry. GEN strains were found to be highly homozygous displaying only 0.062% and 0.066% heterozygosity across the entire genome. Although genetic distance was low between the two isolates, some structural variation was identified, suggesting these are likely descended from the same individual.

Phylogenetic placement of GEN12d2 and GEN58f3 using aligned single copy orthologs (n = 691) was carried out against a background of diverse publicly available genome assemblies (S1 Table) and genome assemblies of representatives for AdEA1, AdEA2 and AdEA3 was carried out. This revealed that both GEN12d2 and GEN58f3, diverge from a common ancestor near the North America & Japan clade supporting their non-domesticated, non-European origin (S8 Fig). However, placement of GEN isolates may be confounded by admixture. To quantify this, two complimentary approaches were used: i) calculated biallele frequency of segregating sites within SCO alignments to construct a drift tree and ii) investigated gene tree local topology.

Analysis of the drift tree recapitulated the three admixture events from basal isolates (Migration weight: AdEA1 = 0.13, AdEA2 = 0.08, AdEA3 = 0.39), however ancestors of the GEN population were predicted to be the donor lineages for the AdEA2 and AdEA3 admixture events (Fig 3A). GEN appeared to share ancestry with the European domesticated ferments (Beer2 and European Wine), however this was only observed in drift trees with >13 migration edges (Migration weight = 0.025) (Fig 3A). Local topology was then investigated to identify the nearest neighbour to the GEN isolates for each gene tree (Fig 3B). Although GEN could not be resolved in reciprocal monophyly with a single other clade across most gene trees (44.9%), 12.4% was associated with North America & Japan isolates as their nearest neighbour. A further 29.5% of gene trees were associated with one of the basal clades as the nearest neighbour. Interestingly, GEN isolates were closest to the representative AdEA3 individual across 6.6% of gene trees further supporting a GEN ancestor as the donor lineage of the AdEA3 admixture event. Only 2.5% of gene trees had domesticated European wine and Beer2 clade isolates as the nearest neighbour (Fig 3B), though the low frequency of this topology could not be disentangled from incomplete lineage sorting. Taken together, these two analyses suggest that the GEN lineage is related to an ancestor of the North America & Japan clade, has limited evidence for admixture with European domesticated isolates and is likely from the same lineage as the donor for the AdEA3 and AdEA2 admixture events.

### Recombination between commercial lineages drives spontaneous isolate genome diversity

Admixture content derived from basal, non-wine isolates that are not found within spontaneous grape ferments provides evidence that spontaneous isolates have undergone feralization, deriving at least some of their genetic content from outside the local winery microbial community. However, the extent to which these spontaneous wine isolates represent ‘clones’ of commercial starter or non-commercial wine isolates remains unknown. To determine this and to understand the genomic background of spontaneous wine strains, genome wide identity by state (IBS) calculations were performed between all pairwise comparisons of both WRP and spontaneous wine isolates. While a small subset of spontaneous isolates (n = 28) were genetically identical to a specific wine reference strain, most were distinct (S9 Fig). Permutation based clustering (n perm = 50000) of spontaneous isolates identified 111 groups which contained 23 putative clonal clusters (S10 Fig and S3 Table). Spontaneous IBS clusters were observed across multiple years and between locations (S9 Fig). Lack of population structure between wineries suggests that spontaneous yeast are actively being transferred out of the immediate winery environment with drift occurring between wineries. This was further supported by principal component analysis (PCA) (S6 Fig) with admixed spontaneous isolates showing low (<0.90) IBS with WRP isolates.

Higher heterozygosity rates were observed within individual WRP isolates compared to the spontaneous isolates in this study (S11 Fig), with many spontaneous strains showing extremely low heterozygosity, which may be due to genome renewal [30,31]. While spontaneous isolates did not represent clonal lineages of WRP strains, they could represent recombined genotypes from within the WRP genetic pool. Estimates of linkage disequilibrium (LD) decay between the spontaneous and WRP sets were used to determine if significant sexual recombination was occurring within the spontaneous population. The rate of LD decay was divergent between spontaneous and WRP isolates (S12 Fig) with low levels of LD decay observed in WRP isolates while rapid decay of LD was observed in the spontaneous wine isolates. Separating the WRP isolates based on the clade assignments of [16], revealed similar LD decay values between the Vin7 (n = 3), PdM (n = 23) and Mixed Wine isolates (n = 60). WRP isolates therefore appear stable, with little sexual recombination, while the spontaneous populations display evidence for extensive recombination.

**Fig 4:**
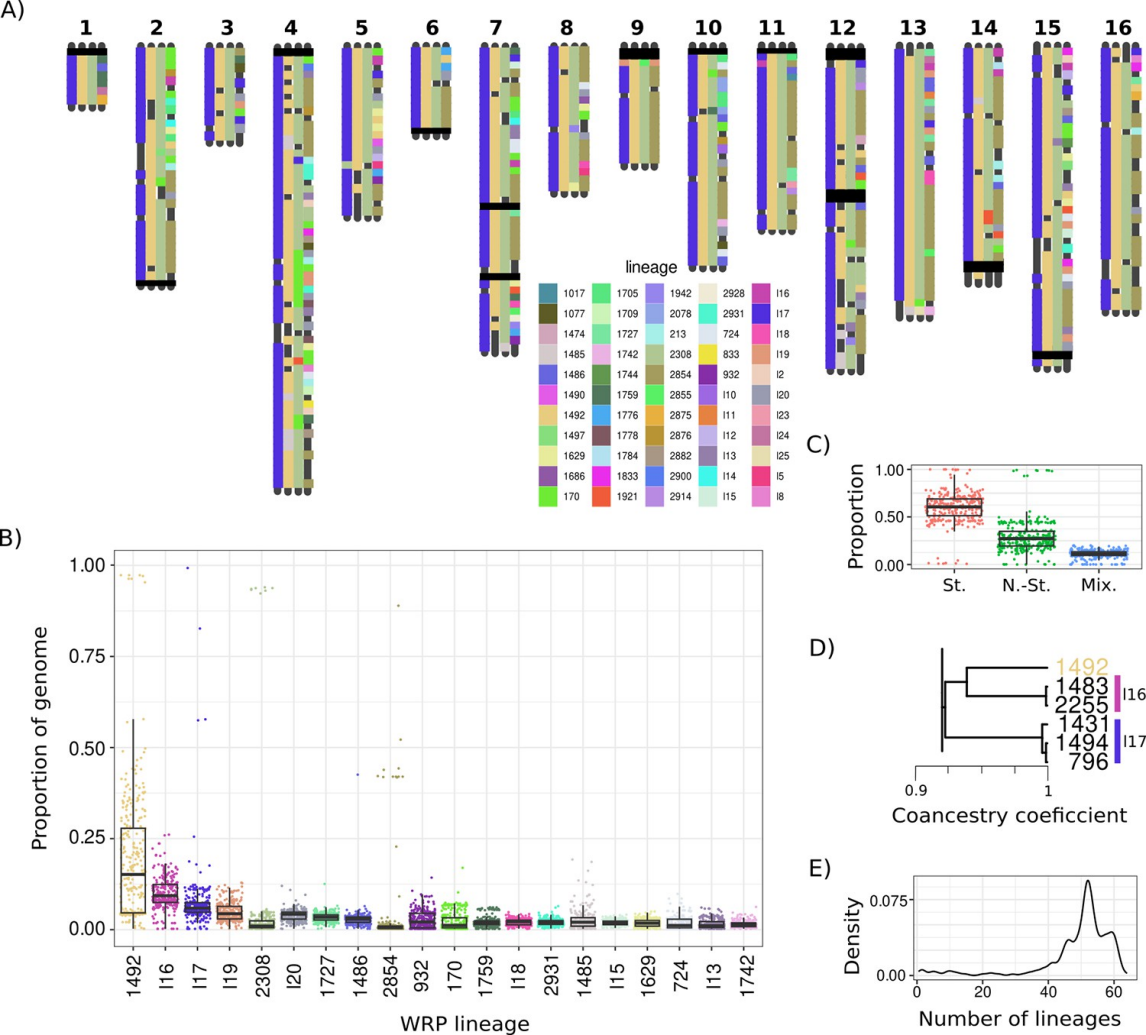
Contribution of WRP strain genetic content to near-homozygous spontaneous isolate genomes. A) Window-wise 25kb Identity by State calculated for all pairwise comparisons of WRP and near-homozygous diploid spontaneous isolates with only select isolates shown. Colors indicate assigned WRP strain lineage of origin ranging from near identical to highly mixed. Block-wise assignment to chromosomes can be found above the figure. An extended figure containing all near-homozygous diploid spontaneous isolates can be found in S11B Fig) Top 20 WRP lineages that contribute genomic blocks to spontaneous isolates and their proportion contribution to each spontaneous isolate tested based on 25kb windowed IBS. Boxes showing the first and third quartile range (IQR) while whiskers extend to a maximum of 1.5 * IQR. C) Proportion of spontaneous isolate genomes with assigned Wine Reference Panel lineages within Starter (St.), Non-Starter (N.-St.), and Mixed (Mix.). Boxes showing the first and third quartile range (IQR) while whiskers extend to a maximum of 1.5 * IQR. D) Dendrogram showing the co-ancestry relationship between the top three lineages, excised from S9E Fig) Density plot showing the number of WRP strain lineages that contribute to the genome of lowly heterozygous spontaneous isolates.

To determine if the extensive LD decay in spontaneous strains was due to recombination primarily between a set of WRP strains, local haplotype origin was investigated across a set of near homozygous spontaneous isolates (genome wide heterozygosity ≤ 0.05%, n = 292). First, hierarchical clustering of the pairwise co-ancestry coefficient (f_(AB)_) between strains was used to reduce the WRP dataset into 24 lineages and 62 unique isolates (S13 Fig, S4 Table). IBS was then calculated across the spontaneous genomes using maximum IBS to identify the most probable WRP lineage for 10, 15, 20 and 25kb windows across each spontaneous genome. The minimum IBS cutoff for a putative match was set at 0.98 to remove any divergent matches that are absent from the WRP set. All window sizes showed a similar ancestry distribution (S14 Fig), therefore the largest (25kb) was selected for further analysis. Spontaneous isolates had an average of 384 (95% CI: 381–388) 25kb windows assigned to WRP origin with a median IBS of 0.994 and only 16 windows having too few sites (>20% missing). Although a small number of spontaneous isolates were near clones of WRP strains (n = 8, S5 Table), the vast majority (99%) could be assigned a mixed WRP ancestry, suggesting that gene-flow and meiotic recombination between WRP starter haplotypes occurs frequently in the environment and that most spontaneous yeast derive through feralization of domesticated strains (Figs 4A and S15). Spontaneous yeast isolates with mosaic ancestry are therefore referred to as feral for the remainder of the analyses.

Most feral isolates were comprised of genetic contributions from many WRP strains and 96% of WRP lineages/isolates contributed genetics to at least one window of a feral isolate genome (Figs 4B and S14). Partitioning the WRP lineages into three categories WRP starter (n = 40), non-starter (n = 39) and lineages containing both starter and non-starter isolates (mixed n = 7) revealed that feral isolates derive a mean proportion of 0.6 (95% CI: 0.58–0.62), 0.29 (95% CI: 0.27–0.31) and 0.11 (95% CI: 0.106–0.114) from each category of WRP lineages (Fig 4C). However, one commercial strain 1492 (Enoferm CSM, Lallemand) contributed greater than 0.2 of the genome (95% CI: 0.18–0.22) to 118 isolates out of the 292 tested (Fig 1B). Furthermore, lineage l16 (Lalvin ICV D254, Lallemand; Uvaferm HPS, Lallemand) contributed an average of 0.12 and shared a close putative ancestor with 1492 (f_(AB)_ ~ 0.94) (Figs 4D and S13). This pattern was observed even when considering a single isolate per spontaneous putative clonal cluster, demonstrating that these two related lineages are contributing to the genetic landscape far more than other WRP isolates. Mosaic feral genomes ranged from slightly (≤ 5 lineages) to highly mixed (≥ 40 lineages) and the genome of each feral strain was derived from a median of 42 different WRP lineages/isolates (Fig 4E). Despite this, many loci failed to be assigned to a sampled WRP isolate at the 0.98 IBS cut-off. When considering known admixture loci from the Beer2 clade and basal lineages, few windows remained unassigned which may be the result of gene-flow from divergent wine strains outside of the WRP utilized in this study.

### Spontaneous yeast exhibit high levels of genome instability that is associated with admixture

Whole genome duplications and aneuploidies have been shown to occur in yeast in response to biotic or abiotic stress and are a hallmark of adaptation to human-associated environments, such as wine and beer production [32,33]. To investigate this, genome-wide ploidy was estimated using frequency bias of biallelic 21-mers for each spontaneous and WRP isolate utilized in this study. 21-mers were then grouped by allelic frequency pattern, ie. the proportion of biallelic kmers that exhibit AB vs AAB/AAAB/AAAAB frequency, to determine genome wide ploidy with SmudgePlot [34]. This revealed that the majority of both spontaneous and WRP yeast were diploid, while polyploidy occurred at similar frequencies in both spontaneous isolates (8.3%) and their wine reference panel (7.5%) counterparts (S16 Fig, S6 Table).

To investigate the level of aneuploidy within spontaneous isolates read depth across each called genotype was calculated for isolates with average genome wide coverage greater than or equal to 20X, identifying 334 spontaneous isolates for further analysis. Chromosomes displaying aneuploidy were predicted if the chromosomal depth distribution had a ≥0.5 rank biserial effect size when compared to all other chromosomes based on the results of a Wilcoxon rank-sum test. Power analysis was carried out on this method through *a priori* simulation of polysomic and monosomic chromosomes while accounting for multiple aneuploidies and partial chromosome duplication/loss (S17 and S18 Figs,; see methods for further details).

**Fig 5:**
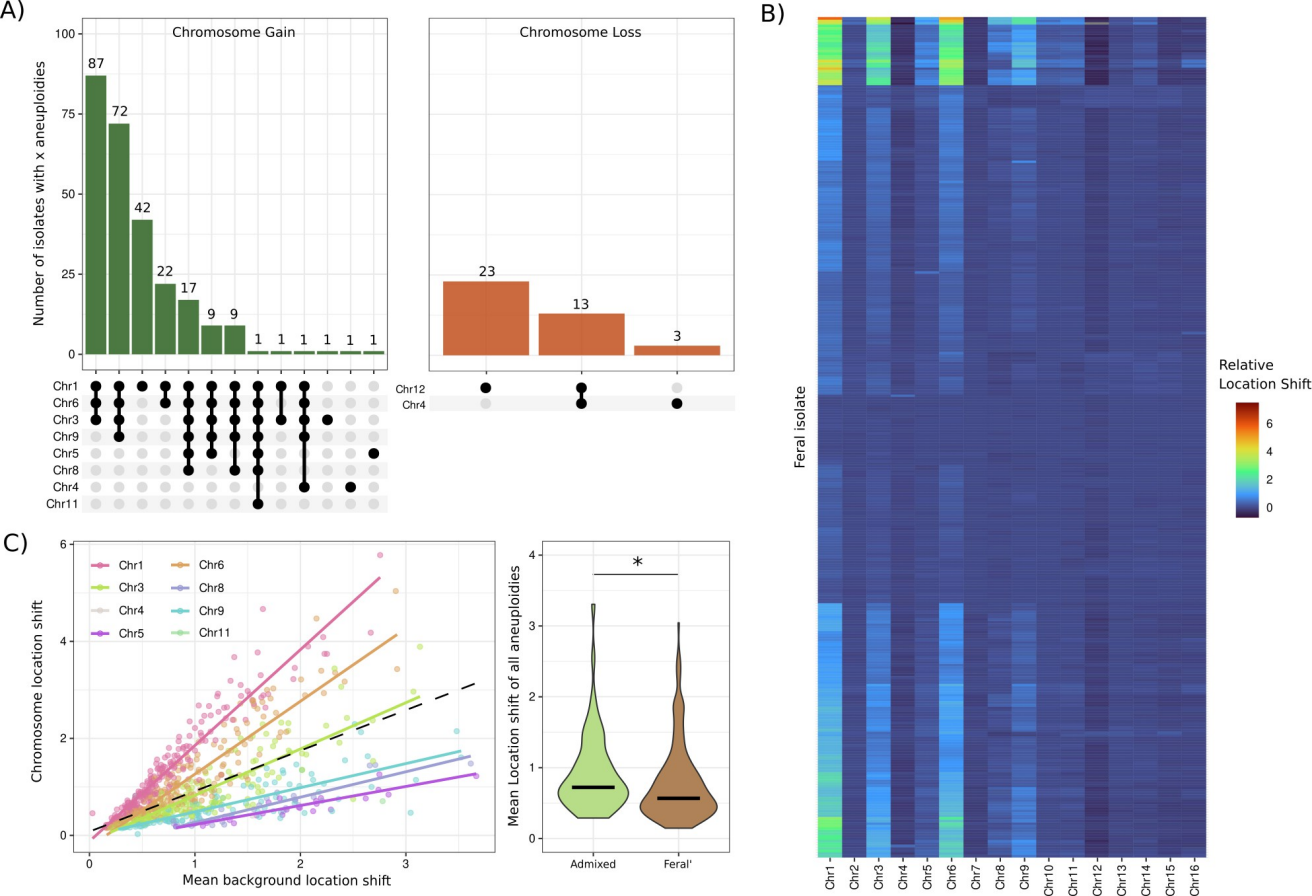
Genomic instability is widespread in spontaneous yeast and is associated with admixture. A) Chromosome gain (left) and chromosome loss (right) events observed in spontaneous isolates. Upset plot nodes represent aneuploidies that were present simultaneously in the same individual along with the number of times each pattern was observed shown above bars. B) Hodges-Lehmann estimate of location shift for each spontaneous isolates clustered using the Ward D2 method. Shift values represent estimated location shift in read depth distribution across each chromosome divided by the median background read depth. Therefore, shift values represent gain or loss of chromosomal read depth against the background distribution, i.e. 0.5 shift per chromosome duplication. C) Determining if chromosome copy number is predictive of general genome instability. Left: Linear regression of per isolate chromosomal location shift against mean background location shift, ie. the average location shift of all chromosomes excluding the chromosome on the y-axis. Right: per isolate mean location shift for isolates with admixture (AdB1, AdEA1, AdEA2, and AdEA3) and all other spontaneous isolates that lacked admixture (Feral’). Median values are shown as a black line within the violin. Statistical significance was calculated between the two distributions using a Mann-Wittney U-test (pvalue = 0.044) to determine if the probability of a random observation from Admixed (number of isolates in test = 90) is greater than a random observation from Feral’ (number of isolates in test = 244) is larger than the contra.

Spontaneous wine isolates displayed a high frequency of aneuploidy, with 79.04% of assessed strains (Fig 5A, S7 Table) containing at least one chromosomal aneuploidy, compared to only 13.2% in WRP strains (S19 Fig and S8 Table). Chromosomal gain events (Fig 5A; left) were far more frequent than loss (Fig 5A; right), comprising 94.4% of all spontaneous aneuploidies. A clear negative correlation was also observed between chromosome length and the frequency of chromosomal gain (S20 Fig). Most gain events involved chromosomes 1, 3, 6, and 9; with a trend towards multiple aneuploidies being observed within single isolates (Fig 5). In contrast, chromosomal loss events were infrequent and almost exclusively involved the longer chromosomes (Fig 5A), aligning with previous results from natural populations [8] and artificial selection experiments [33,35].

Polysome copy number (the number of chromosomes gained through aneuploidy) in spontaneous isolates was highly variable suggesting that the spontaneous genomes are unstable (Fig 5B). The Hodges-Lehmann estimate of location shift was used to estimate the relative change in read depth distribution between polysomes and the 2N background. Location shifts were then divided by the 2N median read depth to estimate copy number variability within isolates (Fig 5B). This revealed that a large subset of spontaneous isolates were highly polysomic containing multiple polysomes with high estimated location shift corresponding to a ~8-15N gain per polysome. In stark contrast, diploid diverse yeast isolates (from Fig 1A) with ≥ 20X coverage showed at most ~1-2N change per polysome when aneuploidies were present (S21 Fig). Furthermore, aneuploid copy number did not cluster according to the topology of the whole genome phylogeny (Fig 1A), demonstrating that high copy number occurred independently rather than being associated with a particular genetic background.

A subset of highly polysomic spontaneous isolates were inspected for chromosome normalized read depth distributions that align with chromosome gain rather than structural variation (S22 Fig) and compared to 2N non-aneuploid isolates (S23 Fig). Polysomes with exceptionally high location shift were found to be largely congruent with normalized chromosome read depth. Yet, location shift appeared to slightly underestimate copy number due to the high level of co-occurring polysomes in spontaneous isolates (S24 Fig, see methods for discussion of simulations). Many chromosome distributions were polymodal suggesting duplication and deletion frequently occurs in high copy number polysomes. Due to these confounding factors, calculation of exact copy number from the read depth data was not carried out, rather location shift is used as a proxy for relative chromosome copy number.

In addition to the frequency of duplications and deletions in highly polysomic isolates, spontaneous yeast genomes were found to be highly unstable with a clear positive correlation between relative location shift and the average location shift of other polysomes (Fig 5C). Polysome location shift was directly proportional to the background copy number of other polysomes present within single isolates for all observed polysomic chromosomes (slope = 0.89, r^2^ = 0.43; Fig 5C dashed line). This shows that isolates with polysomes are more likely to continue to develop chromosome gain events proportional to chromosome length, with the shortest chromosome (Chr1) showing the highest rate (slope = 1.97, r^2^ = 0.91.6; Fig 5C). Furthermore, by using average location shift across all polysomes within single isolates as a proxy for general genome instability, polysomes in admixed strains (AdB1, AdEA1, AdEA2, AdEA3) was significantly (Wilcoxon exact test p-value = 0.044) associated with higher average polysome copy number compared to non-admixed (Feral’) polysomes (Fig 5C) suggesting admixture from divergent isolates has a negative effect on genome stability.

## Discussion

Spontaneous grape fermentations derive *S*. *cerevisiae* yeast from the surrounding environment, rather than starter cultures. Yet, little is known about the genetic diversity present within these natural populations. In this study the genomic landscape and architecture of yeast isolated from spontaneous grape fermentations in Australia was investigated against a reference panel of both starter and non-starter wine strains (Wine Reference Panel; WRP). While it was found that Australian spontaneous yeast were derived from feralized, highly recombined mixtures of multiple distinct WRP lineages, genome instability and inter-clade admixture from beer or non-domesticated lineages potentially of Oceanic origin were found to be common.

Whole genome and chromosome duplication is a key strategy *S*. *cerevisiae* utilizes to rapidly adapt to novel stressors [36–40]. Due to *S*. *cerevisiae*’s selection for the fermentative environment, starter wine strains may not be fit in the natural environment and need to adapt to survive outside of the wine environment. We found that whole genome duplications were observed at similar rates between spontaneous yeast and WRP isolates, yet chromosomal instability (aneuploidies) was far more common in spontaneous yeast. Previous work [33] found a negative correlation between chromosome length and aneuploid frequency. We recapitulate this result, however, relative frequency of chromosome polysomy was different between our study and previous work [33] which may be due to unique stressors of the fermentative to spontaneous to fermentative environmental transfer. This study also found that gain of chromosomes 1, 3, 6, and 9 was common, agreeing with past studies [8,33,41], where the increased copy number of these chromosomes has been implicated in responding to drug [38], temperature [42], and environmental [33,43] stress. The statistical framework utilized in this study to identify aneuploidies also had the ability to estimate relative copy number using the Hodges-Lehmann estimate of location shift. Spontaneous isolates with aneuploidies were found to have high levels of genomic instability with widespread duplications and insertions along polysomes, that is absent from previously sequenced isolates from other lineages and previous reports of yeast from spontaneous ferments [3,44]. Copy number of polysomes was directly proportional, in a linear fashion, to the copy number of other co-occurring polysomes. It was shown that this admixture played a role in genomic instability which may further increase adaptability in isolates of mixed ancestry. Alternatively, aneuploidy and by extension genomic instability may be utilized as a compensatory mechanism to recover from admixture of maladaptive alleles or loss of beneficial alleles.

Previous work [8] has suggested that feral *S*. *cerevisiae* strains show higher rates of heterozygosity than their European wine counterparts. European wine clade isolates had higher heterozygosity than spontaneous samples in this study, despite there being clear signs of sexual reproduction between spontaneous isolates. One explanation for this conflicting observation comes from a genetic study of yeast derived from spontaneous wine fermentations, whereby heterozygous isolates transitioned into homozygous diploids through genome renewal [30,31]. Alternatively, inbreeding rates may be increased in a winery environment between ferments where transference between clusters of related individuals may be infrequent. This may have caused us to underestimate the rate of heterozygosity, in spontaneous isolates and future studies should investigate heterozygosity in environmental isolates.

Unlike beer, where fermentations are conducted year-round, with yeast reused across multiple ferments through pitching, wine production is seasonal and requires wine yeast to find suitable refuge between vintages. This forces *S*. *cerevisiae* to escape into the broader environment [4,23,24], where they are subjected to novel selection pressures [15,45]. The lack of population structure observed between years and wineries suggests that there is transference between wineries, although it was not possible to directly measure the winery microbial community that persisted between ferments versus those that have escaped into the local environment and returned. As wine strains exhibit decreased interbreeding though low spore viability [3,46], it was expected that spontaneous yeast genomes would be comprised of haplotypes derived from few recombination events between WRP isolates. Previous work on the genetic origins of yeast within spontaneous wine ferments in New Zealand and Canada [44,47] investigated general population structure between yeast isolates finding that the majority of samples fell within the European wine clade with few closely related outliers. Canadian populations contained a genetically distinct clade of Pacific West Coast Wine yeast in reciprocal monophyly with the European wine clade [44]. Investigations for population mixture revealed that separation from the European wine is due to admixture with the American Oak population. In the present study, by using fine scale phylo- and population genomic analyses to comprehensively assign local genomic windows to their most likely lineage of origin, it appeared that spontaneous populations contained both near-clonal starter and highly mosaic isolates with a small number of related starter lineages contributing most loci. Though it was not possible to determine if this occurred through biotic (e.g. selection for higher sporulation rates) or abiotic (e.g. increased usage) means, the majority of genetics present within recombined spontaneous isolates was derived from highly utilized commercial starter products. This suggests that abiotic forces such prolonged use of the same monoculture starter strains likely plays a significant role in the genetics that are present within spontaneous wine fermentations. These findings suggest that the use of commercial starter strains in high frequency may decrease genomic diversity within spontaneous ferment communities. This may be especially pertinent in countries, such as Europe and Asia, where native, divergent *S*. *cerevisiae* populations are widespread, potentially causing the standing variation to be overrun with few highly used commercial lineages through drift. Thereby decreasing any effect microbial terroir may have on ferment characteristics.

Admixture between *S*. *cerevisiae* clades [4] has been an important source of adaptive novelty resulting in the origin of genetically and phenotypically distinct strains such as the Beer2 clade [19]. We observed three distinct admixture events between diverse non-domesticated isolates and wild Australian samples, which may be driving strain diversity. This suggests that isolates derived from spontaneous ferments are escaping into the local environment where they then have the opportunity to mix with other divergent lineages. Phylogenetic reconstruction of each admixed locus identified that gene-flow was unique from those previously observed in Beer and Lager/Ale [19,20]. Investigation into heterozygosity around admixed loci suggested that backcrossing with wild isolates is ongoing at most admixed loci. Yet, many loci have been retained across all years, despite others being removed, which may represent adaptive introgression, a phenomenon that has been observed in many species, providing novel genetic variation to respond to selection [48–50].

Oceania is thought to lack native *S*. *cerevisiae* populations, with these only being described after wine and beer production began in the late 19^th^ century [25]. In contrast, evidence exists for Indigenous Australians carrying out fermentation utilizing non-*Saccharomyces* yeast lineages [26]. Presence of admixed samples presents a clear conundrum—where did the source lineages originate and how did they come to be present in Australia? The simplest explanation is that domesticated Asian yeast were introduced through fermentation of Sake and Huangjiu in Australia. Yet, local phylogenetic reconstruction revealed that branches leading to the three admixture events all occur before the most recent common ancestor of domesticated Sake, Baijiu, and Huangjiu suggesting donor lineages diverged pre-domestication. Endemic lineages of *S cerevisiae* are widespread across Southeast Asia [4,8] and recent work isolated an individual from the China (CN) X lineage in New Caledonia [4], an island in the South Pacific approx. 1,100 km east of Australia. Although these are genetically dissimilar to either admixture source lineage, they collectively suggest that the dispersal history of non-domesticated lineages may be far more complex and widespread than previously thought. Further supporting this, two samples isolated from wild niches in Northern Australia share a common ancestor with the donor of the AdEA2 and AdEA3 introgression events, which were isolated from Southern Australia. The geographic separation of these isolates suggests wild pre-domestication lineages are widespread in Australian contemporary populations which may predate colonisation.

Taken together these results show that *S*. *cerevisiae* cultured from spontaneous fermentations exhibit high levels of genome instability and are affected by admixture, with gene flow from multiple independent lineages that has been maintained at fixation across multiple years. Despite this, closely related starter lineages contribute the majority of genetics to the assessed isolates, providing evidence for the effect commercial monoculture use may have on the genetic diversity of ferment derived microorganism populations. Finally, these results show that non-European derived, non-domesticated lineages of *S*. *cerevisiae* are present in Australian ecological niches and are actively admixing with feralized domesticated isolates that innoculate spontaneous wine ferments.

## Materials and methods

### Yeast strain selection and sequencing

Extracts from industrial level spontaneous fermentations were sourced from wineries in three wine regions near Adelaide, South Australia (S9 Table) during the 2014, 2016, 2018 and 2019 calendar years. Industrial level spontaneous ferments were carried out from non-sterile crushed grape juice/must and sampled at different times throughout the ferment based on remaining sugar content. Fermentations were uninoculated, i.e. without starter, instead allowed to accrue microbes from the surrounding environment which have been known to be carried by soil, grapes and insects [7,21,23,24]. Extracts were plated on WL media containing 25 μg/mL chloramphenicol and then arrayed on YPD media +25 μg/mL chloramphenicol in 96-well format using a PIXL automated colony-picker (Singer Instruments) or by hand. Individual colonies were identified using high-throughput ITS sequence analysis [26]. Isolates identified as *Saccharomyces cerevisiae* were cultured in liquid YPD, with DNA extracted using the Gentra Puregene Yeast/Bact. Kit (Qiagen). Whole-genome sequencing was performed using the Illumina Nextera XT library protocols and sequenced at 2 x 300bp read length on the MiSeq platform (Ramaciotti Centre for Functional Genomics, Randwick, Australia).

Short read data were input into jellyfish to construct 31-mer databases for each sample. Jellyfish dump was used to generate canonical 31-mers for each sample with a minimum number of observations greater than 4. Canonical 31-mers were queried against databases for each *Saccharomyces sensu stricto* species with an available reference genome (*S*. *cerevisiae* [27], *S*. *paradoxus* [51], *S*. *mikatae* [52], *S*. *kudriavzevii* [52], *S*. *jurei* [53], *S*. *arboricous* [54], *S*. *eubayanus* [55], and *S*. *uvarum* [56]). This produced a presence/absence marker dataset for each sample against each *sensu stricto* reference genome. Markers for each sample were then filtered to remove markers that were present in more than one *sensu stricto* reference database, allowing for estimates of the proportion of genomic content from each *sensu stricto* species to be calculated.

Strains GEN12d2 and GEN58f3 were originally isolated from environmental samples (plant and feather sample respectively) collected from Bray Park, Queensland. Environmental samples were cultured in liquid YPD with 5% ethanol and 25 μg/mL chloramphenicol. Subsamples were then grown on WL + 25 μg/mL chloramphenicol and colonies were selected by hand and grown in YPD liquid. These selected individual colonies were identified using high-throughput ITS sequence analysis [26]. Isolates identified as *S*. *cerevisiae* were cultured in liquid YPD and DNA was extracted by lysis of protoplasts through digestion with zymolase and potassium acetate [57]. Nanopore sequencing libraries were prepared using the SQK-LSK112.24 kit and loaded into a FLO-MIN112 (R10.4) flow cell. Fast5 files were base called and demultiplexed using Guppy v6.4.8 (Oxford Nanopore Technologies, Oxford, UK) with the ‘sup’ model and a minimum quality score filtering of 7. A total of 91X and 119X coverage was obtained for strains GEN12d2 and GEN58f3, respectively.

### Quality control, mapping and genotyping

Multiple publicly available WGS resequencing datasets were used in this analysis containing wine [16] and non-wine [3, 6–8] isolates. SRA accessions can be found in S1 Table. Raw fastq data was quality controlled using fastqc (https://www.bioinformatics.babraham.ac.uk/projects/fastqc/) and ngsReports v2.2.3 [58]. Samples that showed detectible levels of adapter contamination were trimmed using Trimmomatic v0.3.9 [59]. Reads were then mapped to the S288C *S*. *cerevisiae* reference genome [27] using NextGenMap v0.5.5 [60] under default settings to reduce reference mapping bias [61,62]. Aligned reads were then sorted, indexed and filtered (MAPQ>20) using SAMtools v1.6 [63] and had duplicates removed using Picard MarkDuplicates (https://github.com/broadinstitute/picard) before being genotyped individually and merged by BCFtools v1.17 [64]. Sample genotypes with GQ ≤ 20 or DP ≤ 6 were set to missing (./.) and then sites were removed if more than 80% of sample genotypes were missing. This produced the filtered genotype dataset used throughout the paper. To carry out local phylogenetics a second phased genotype set was made. This included additional samples from other diverse clades, see S1 Table for accessions. Genotype phasing was carried out using SHAPEIT2 v2.r837 [65] under default settings without imputation. Both the filtered and phased VCF records were then converted to Genomic Data Structure (GDS) format using SeqArray v1.4.0 [66] for downstream analyses.

### Genome assembly

De-novo genome assemblies for *S*. *cerevisiae* strains GEN12d2 and GEN58f3 were performed using Canu v. 2.2 [67]. A consensus sequence for each assembly was obtained using Medaka v1.7.3 (Oxford Nanopore Technologies, Oxford, UK).

[68][27][64][64]Short read genome assembly was carried out for a subset of the MO samples and AdEA1, AdEA2, AdEA3 populations (S10 Table) using Velvet v1.2.10 [69] under default settings. The S288C gene annotations [70] were then lifted over onto short read, longread and publicly available genome assemblies (S10 Table) using Liftoff v1.6.3 [71].

### Calculation of identity by state, coancestry coefficient and principal components

The filtered GDS was passed to geaR v0.1.0 [72] to construct windows (10, 15, 20 and 25kb), with repeat regions excluded, and calculate nucleotide diversity across each wild genome to identify isolates with low levels (<0.5%) of heterozygosity. Identity by state for each pairwise comparison of WRP and spontaneous wine yeast was calculated at the genome wide level with SNPrelate v1.32.0 [73]. Genome wide calculation of the coancestry coefficient and Z scores were calculated by passing IBS values to the snpgdsCutTree function in SNPrelate v1.32.0 [73] with 50000 permutations and visualized using snpgdsDrawTree. The dendrogram was then manually inspected for clusters using a Z-score cut-off of 3 and an IBS of 0.99 to identify lineages. To identify WRP origin of wild loci, 10, 15, 20 and 25 kb tiled window analysis was then carried out by first partitioning non-repetitive variants into windows using gear v0.1.0 [72] then passed to SNPrelate v1.32.0 [73] for IBS calculation. Pairwise comparisons of spontatenous vs WRP isolates were then carried out to identify the highest IBS values for each spontaneous sample across windows at a 0.98 IBS cut-off. Genome wide contributions were then resolved into the lineages identified above to robustly determine WRP origin. Principal component analysis was carried out on the filtered genotype GDS file using SNPrelate v1.32.0 [73] and each principal component pair was visually inspected for stratification of spontaneous isolates.

### Phylogenetics and population genomics

Linkage disequilibrium was calculated using the PopLDdecay tool v3.42 [74] under default settings. BUSCO v4.1.2 [75] was used to identify 1386 single copy orthologs in the S228C annotation [70] and genotypes (including invariant sites) were extracted within SCOs using gear v0.1.0 [72] from the filtered genotype GDS. Gene trees were then reconstructed using IQ-TREE v2.2.2.3 [76] allowing for best model inference (-m MFP) (best-fit substitution models for each window can be found in S11 Table). Gene trees were then passed to ASTRAL-III v3.0 [77] to estimate the species phylogeny under default settings. ADMIXTURE v1.3.0 [78] was used to reconstruct the ancestral population structure at 2–5 values for the number of *a priori* populations (k). TreeMix v1.13 [79] was used to identify admixture from non-wine *S*. *cerevisiae* clade into outliers identified using PCA, topology and ADMIXTURE. Data was converted from GDS to tab separated allele frequency values as required by TreeMix using the R programming language. To further investigate these regions, within population/individual nucleotide diversity (Nei’s *π*) [28], between population/individual absolute genetic distance (Nei’s *d*_*XY*_) [28], and $\hat{f_{d}}$ [29] were calculated using the geaR v0.1.0 R package [72] across 10kb tiled windows containing non-repetitive regions.

As the source population could not be utilised to identify incongruent topologies a subtree sorting method was implemented, inspired by the quartet subtree approach for comparing topologies [80] and TTD [62], to test local topology in the R programming language. Phased genotypes, that were not within repetitive regions, of all samples utilized in the whole genome tree (wild (n = 411), WRP (n = 169) and diverse strain origin (n = 91)) were extracted in 10kb windows and output to *fasta* with separate sequences for each haplotype using gear v0.1.0 [72]. Maximum likelihood phylogenies were then reconstructed for each window using IQ-TREE v2.2.2.3 [76] utilizing ModelFinder Plus (-m MFP) to estimate the best fit substitution model for each alignment (best-fit substitution models for each window can be found in S12 Table). Local phylogenies were tested for incongruence by iterating across all admixed isolate haplotypes and extracting a subtree containing one admixed isolate haplotype, all individuals in the closest clade to the source of admixture (AdEA1:Baijiu&Huangjiu; AdEA2:NA&Japan; AdEA3:NA&Japan), Mediterranean oak isolates and WRP wine isolates with PhyTools v1.5 [81]. As Mediterranean oak populations have been shown to share a common ancestor with European wine yeast after the dispersion of yeast out of Asia [7], haplotypes that resolved in paraphyly with this MRCA can be considered incongruent. Local phylogenies with subtrees that were incongruent (i.e. at least one wild haplotype was both monophyletic with a source population and paraphyletic with all WRP and Mediterranean Oak isolates) were treated as evidence for admixture.

Phylogenetic reconstruction of admixture source lineages was carried out by selecting loci with greater than or equal to three consecutive, fixed 10kb windows flagged by $\hat{f_{d}}$ [29] and/or local topology as introgressed. As no loci were fixed in AdEA2 a single homozygous diploid isolate from pop AdEA2 was used (Q-3_S92). Windows at admixture breakpoints, ie the first and last 10kb windows, were then discarded as these may contain recombinant genotypes from both ancestries. Phylogenetic reconstruction was then carried out using IQ-TREE v2.2.23 [76] by selecting the best fit substitution model (-m MFP) for each window (best fit models can be found in S13 Table). All trees were then concatenated and passed to ASTRAL-III v3.0 [77] along with lineage assignments (S1 Table) to collapse clades into their respective lineages. As Astral-III can utilize local trees where not all leaves in the species tree are present, a single species tree was constructed incorporating all three East Asian admixture events even though they lacked homozygous overlapping introgression windows.

Whole genome phylogenetic reconstruction was carried out on genome assemblies by building individual gene trees from multiple sequence alignments carried out with MUSCLE v5.1 [82] for each of the 691 SCOs with acceptable gene alignments (≥5 isolates in alignment and ≥80% of the S288C gene length annotated) in IQ-TREE v2.2.2.3 [76] with model selection (-m MFP) (a list of models for each gene use can be found in S14 Table). Gene trees were then passed to Astral-III v2.2.2.3 [77] for species tree reconstruction under default settings. Reconstruction of the drift ancestry of the assemblies was carried out by identifying segregating bialleles within each of the SCOs. Biallele frequency was then calculated and converted to the TreeMix format and drift trees for m = 5–15 were calculated using TreeMix [79].

### Ploidy and aneuploidy estimation

Ploidy estimation was carried out using Smudgeplot v0.2.5 [34] for each WRP and wild sample with k = 21. Per site read depth was extracted from the filtered genotype GDS using SeqArray v1.4.0 [63] and statistics calculated using the R programming language. Samples with less than 20X estimated coverage were removed from the dataset before proceeding with aneuploidy and genome instability analyses.

As individual sequencing reads are expected to sample each position in the genome randomly and in an independent manner [83], aneuploidies were identified by comparing the distribution of per-site chromosomal read depth to the genome wide depth distribution (excluding the test chromosome) using a Wilcoxon rank-sum test. Chromosomes were considered to be aneuploidic if the rank biserial effect size was considered ‘large’ according to [84] ie rank biserial ≥ 0.5. Wilcoxon tests were carried using the wilcoxon.test function in R and rank biserial effect size calculated using the effectsize v0.8.3 R package [85]. To test the behaviour of this method, a gain or loss aneuploidy event (S17 Fig) and segmental duplication of 0–50% (S18 Fig) of chromosome 6 were simulated and tested against a simulated genome with a background of 0–5 co-occurring aneuploidies (S17 and S18 Figs; only 0 and 5 shown). Genome wide read depth distribution was sampled from a normal distribution with mean = 30 and σ = 10. The number of simulated sites for each chromosome were sampled according to the number of sites observed in the empirical dataset. Aneuploidies were simulated in the genome wide background by scaling read depth for 0–5 chromosomes to 3N (ie, 1.5x chromosome read depth). Test chromosomes for aneuplodies were scaled by a factor iterating through -1 to 1 by 0.1 to simulate read depth bias (2N read depth + 2N read depth x scaling factor). Test chromosomes for duplications had a fraction of their length (-0.5–0.5) scaled by a factor of 1.5 (ie –0.5 has 1N for half the chromosome; 0.5 has 3N for half the chromosome). This showed that using a large rank biserial effect size (≥0.5) was robust at identifying true aneuploidies vs sequencing depth variation and segmental hemizygosity.

Location shift, as a measure of polysome copy number, was calculated by estimating the Hodges-Lehmann location shift from each Wilcoxon rank-sum test. Location shift of polysomes was then utilized to test if individual polysome copy number is predictive of overall genomic instability (the degree to which other chromosomes are polysomic). A generalized linear model was fit to each [chromosome location shift] ~ [mean location shift of other polysomes], stratified by genomic chromosome, using the glm function in R. Mean location shift across all polysomes was then used as a proxy for overall levels of genomic instability within each isolate. To determine if there was any significant difference between admixed and non-admixed spontaneous isolates, a Wilcoxon exact test was used to determine if the probability of a random observation from admixed isolates is greater than a random observation from non-admixed isolates is larger than the *contra*.

To evaluate the effect multiple background polysomes with high copy number has on the Hodges-Lehmann location shift four scenarios were simulated. Background polysomes were simulated with 0 (2N), 1 (4N), 2 (6N) and 3 (8N) location shift for 1–5 co-occurring polysomes based on the same method as previous simulations. A single randomly selected foreground chromosome was simulated to have a true read depth shift of 0–5 iterating by 0.5 to represent a 0-10N gain (S24 Fig). This showed that the Hodges-Lehmann estimated location shift was a true representation of copy number when no co-occurring polysomes were simulated. However, in other cases location shift underestimated true copy number in proportion to both the number of co-occurring polysomes and their copy number. In the most extreme test case of 5 co-occurring polysomes with 8N copy number (S24 Fig), location shift of the test chromosome was only 12% lower than the simulated copy number suggesting location shift is a strong metric to determine relative copy number even in highly unstable genomes.

Per chromosome read depth normalization was then carried out on a subset of randomly selected isolates to further investigate the appropriateness of location shift as an estimator for relative copy number. This was conducted by calculating the median of known 2N chromosomes from the effect size analysis above. Median read depth was calculated across 10kb windows sliding by 2kb and divided by the 2N median to normalize chromosome read depth distributions. Read depths were then plot for each chromosome and overlayed with a scale representing theoretical copy number values (0.5 read depth difference from 2N per copy number change). Plots (S22 and S23 Figs) were manually inspected for alignment with the theoretical copy number scale.

## Supporting information

S1 Fig Genome composition of spontaneous wine isolates. Proportion of 31-mers with an exact match to one of the sensu stricto Saccharomyces species S. arboricola (Sa), S. cerevisiae (Sc), S. eubayanus (Se), S. jurei (Sj), S. kudriavzevii (Sk), S. mikatae (Sm), S. paradoxus (Sp), and S. uvarum (Su) for each spontaneous isolate against a database of 31-mers unique to each species.
(TIF)

S2 Fig Genome wide mean read depth mapped to the S288C genome against the proportion of the genome genotyped with red dashed lines showing the cutoffs for sample retention DP>5 and proportion > 0.8. (TIF)

S3 Fig ADMIXTURE ancestral population structure inference with a priori population number set from 2–5 ordered by their phylogenetic relationship shown in Fig 1A. (TIF)

S4 Fig Principal component analysis of spontaneous isolates shows a lack of widespread population structure between locations and grape varieties across eigenvectors (EV) 1–8. PCA outliers used to compare to spontaneous isolates with non-commercial wine ancestry are circled in red.
(TIF)

S5 Fig TreeMix drift trees iterating through number of migration events (m) 5–10 reveals widespread admixture between genetic clades. Migration edges are colored based on the estimated proportion of drift from source to target population. WRP represents the Wine Reference Panel.
(TIF)

S6 Fig 10kb windowed allele frequency of admixed loci in population AdEA1 across the three years it was observed 2016, 2017 and 2018. Blank windows are where the local topology was unresolved.
(TIF)

S7 Fig Coalescent species tree reconstruction of diverse S. cerevisiae populations along with AdEA1, AdEA2 and AdEA3 using fixed admixture loci identified in Fig 3A. As no loci were fixed in AdEA2 a single homozygous diploid isolate from pop AdEA2 was used (Q-3_S92). Node posterior probability is shown. Scale is in coalescent units.
(TIF)

S8 Fig Phylogenetic reconstruction from 691 single copy orthologs places isolates GEN12d2 and GEN58f3 as sharing a common ancestor with the NA & Japan clade. AdEA1, AdEA2 and AdEA3 are included as an indicator that although they contain admixture (~50% of the genome in AdEA3) they are still placed closer to domesticated lineages. Node posterior probability is shown. The scale is in coalescent units.
(TIF)

S9 Fig Heatmap of isolate vs isolate identity by state (IBS) for all Spontaneous and reference isolates annotated based on type, variety, location, and year. VIO = Viognier, PNT = Pinot Noir, SHR = Shiraz, GRN = Grenache, CHR = Chardonnay, CAB = Cabernet Sauvignon.
(TIF)

S10 Fig Clustering of Spontaneous Isolates using premutation (n = 50000) based clustering of the co-ancestry coefficient. Alternation between grey and white represent lineages with individual isolates in their own lineage (outliers) highlighted in red. Purple bars represent individual isolates recognized as clonal clusters. A list of individuals and their lineage and clonal status can be found in S3 Table.
(TIF)

S11 Fig Distribution of per isolate genome wide heterozygosity for WRP and Spontaneous (Spon.) samples. WRP isolates are broken down into their respective clades Wine = mixed European wine clade; PdM = Prise de mousse and Vin7.
(TIF)

S12 Fig Decay of linkage disequilibrium for WRP (PdM, Vin7 and Wine) and Spontaneous populations as a function of r2 values between pairs of SNPs across the genome. Distances where linkage disequilibrium decayed to half its total value are marked by a black point.
(TIF)

S13 Fig Co-ancestry coefficient dendrogram for all wine isolates with black lines showing those isolates considered lineages for IBS analysis. Red isolates are unique isolates that do not form a lineage.
(TIF)

S14 Fig The proportion of windows assigned to the top 20 lineages identified using co-ancestry analysis in S10 Fig. Four different window sizes 10, 15, 20 and 25kb were used to test for bias in window assignment based on window size.
(TIF)

S15 Fig Window-wise 25kb Identity by State calculated for all pairwise comparisons of WRP and homozygous diploid spontaneous isolates. Colors indicate assigned WRP strain lineage of origin. Block-wise assignment to chromosomes can be found above the figure.
(TIF)

S16 Fig Example of heatmaps generated by SmudgePlot for ploidy inference based on k-mers showing both diploid and polyploid isolates. Upper: Spontaneous samples, Lower: WRP samples.
(TIF)

S17 Fig Rank biserial effect size in simulated aneuploidies against genome wide read depth data. Aneuploidies were simulated in the genome wide background by scaling read depth for 0 and 5 chromosomes to 3N (ie, 1.5x chromosome read depth), aneuploid chromosomes were randomly selected from the empirically observed chromosome aneuploidy rate. Test chromosomes were scaled by a factor iterating through -1 to 1 (0N - 4N) by 0.1 to simulate read depth bias (2N read depth + 2N read depth x scaling factor).
(TIF)

S18 Fig Rank biserial effect size in simulated segmental duplications or translocations against genome wide read depth data. Duplications were simulated in the genome wide background by scaling read depth for 0 and 5 chromosomes to 3N (i.e., 1.5x chromosome read depth), aneuploid chromosomes were randomly selected from the empirically observed chromosome aneuploidy rate. Test chromosomes had a fraction of their length (-0.5–0.5) scaled by a factor of 1.5 (i.e. –0.5 has 1N for half the chromosome; 0.5 has 3N for half the chromosome).
(TIF)

S19 Fig Chromosome gain events observed in WRP isolates. Upset plot nodes represent aneuploidies that were present simultaneously in the same individual.
(TIF)

S20 Fig Aneuploidies show a negative correlation with chromosome size. Linear model fit to the number of observations of aneuploidies for each chromosome across the entire spontaneous isolate pool against chromosome size.
(TIF)

S21 Fig Hodges-Lehmann estimate of location shift for diverse isolates of WRP and diverse origin contained within Fig 2A clustered using the Ward D2 method. Shift values represent estimated location shift in read depth distribution across each chromosome divided by the median background read depth. Therefore, location shift values represent gain or loss of chromosomal read depth against the background distribution, i.e. 0.5 shift per chromosome duplication. Figure legend is scaled to be more easily compared to Fig 5B.
(TIF)

S22 Fig Read depth across the genome (normalized to 2N) broken down by chromosome (dark grey, light grey alternation) for randomly selected high polysome copy number spontaneous isolates as estimated by the Hodges-Lehman estimate of location shift. Dashed horizontal lines denote expected copy number driven read depth increases (0.5 per 1N gain).
(TIF)

S23 Fig Read depth across the genome (normalized to 2N) broken down by chromosome (dark grey, light grey alternation) for selected spontaneous isolates without aneuploidy. Dashed horizontal lines denote expected copy number driven read depth increases (0.5 per 1N gain). The y axis is scaled to match S22 Fig.
(TIF)

S24 Fig Hodges-Lehman location shift underestimates true copy number when co-occurring polysomes are present. Hodges-Lehman location shift in simulated polysomes for polysomes shifted by 0–5 by 0.5 (where 0.5 corresponds to a 1N increase in polysome copy number) against genome wide read depth data that has simulated 0–5 background polysomes co-occurring with the test chromosome. Simulated background polysomes had their read depth shifted by a factor of 0–3 to simulate 2N-8N copy number for background polysomes.
(TIF)

S1 Table Metadata of isolates utilized in this study. (XLSX)

S2 Table Windows (S288C genome) with positive evidence for admixture with the observed allele frequency (AF) and fd. (XLSX)

S3 Table Permutation based clustering of spontaneous wine yeast isolates using the coancestry coefficient to identify lineages and clonal clusters. (XLSX)

S4 Table Commercial lineages assigned to lineages using coancestry coefficient. (XLSX)

S5 Table Spontaneous isolates clonally related to commercial isolates and their lineage origin. (XLSX)

S6 Table Spontaneous isolate ploidy and heterozygosity estimated using smudgeplot. (XLSX)

S7 Table Gain/loss aneuploidies observed in spontaneous samples according to the biserial effect size. (XLSX)

S8 Table Gain/loss aneuploidies observed in WRP wine samples according to the biserial effect size. (XLSX)

S9 Table Winery code and wine region of origin. (XLSX)

S10 Table Publicly available short read genomes and those assembled in this study that were utilized to reconstruct the evolutionary history of Australian niche samples. (XLSX)

S11 Table Genes identified as single copy using BUSCO and the Best Model identified using IQ-TREE during gene tree reconstruction. (XLSX)

S12 Table Best Model identified for genomic windows across the S288C genome using IQ-TREE during genome wide local tree reconstruction. (XLSX)

S13 Table Best Model identified for admixed windows using IQ-TREE during local tree reconstruction. (XLSX)

S14 Table Single copy orthologs from genome assembly annotations and the Best Model identified using IQ-TREE to reconstruct the evolutionary history of Australian niche samples. (XLSX)
