# Supplementary figures and images for: Recombination, admixture and genome instability shape the genomic landscape of Saccharomyces cerevisiae derived from spontaneous grape ferments
Source: PLoS Genet. 2024 Mar 22;20(3):e1011223. doi: 10.1371/journal.pgen.1011223 (PMC10990190; doi:10.1371/journal.pgen.1011223)

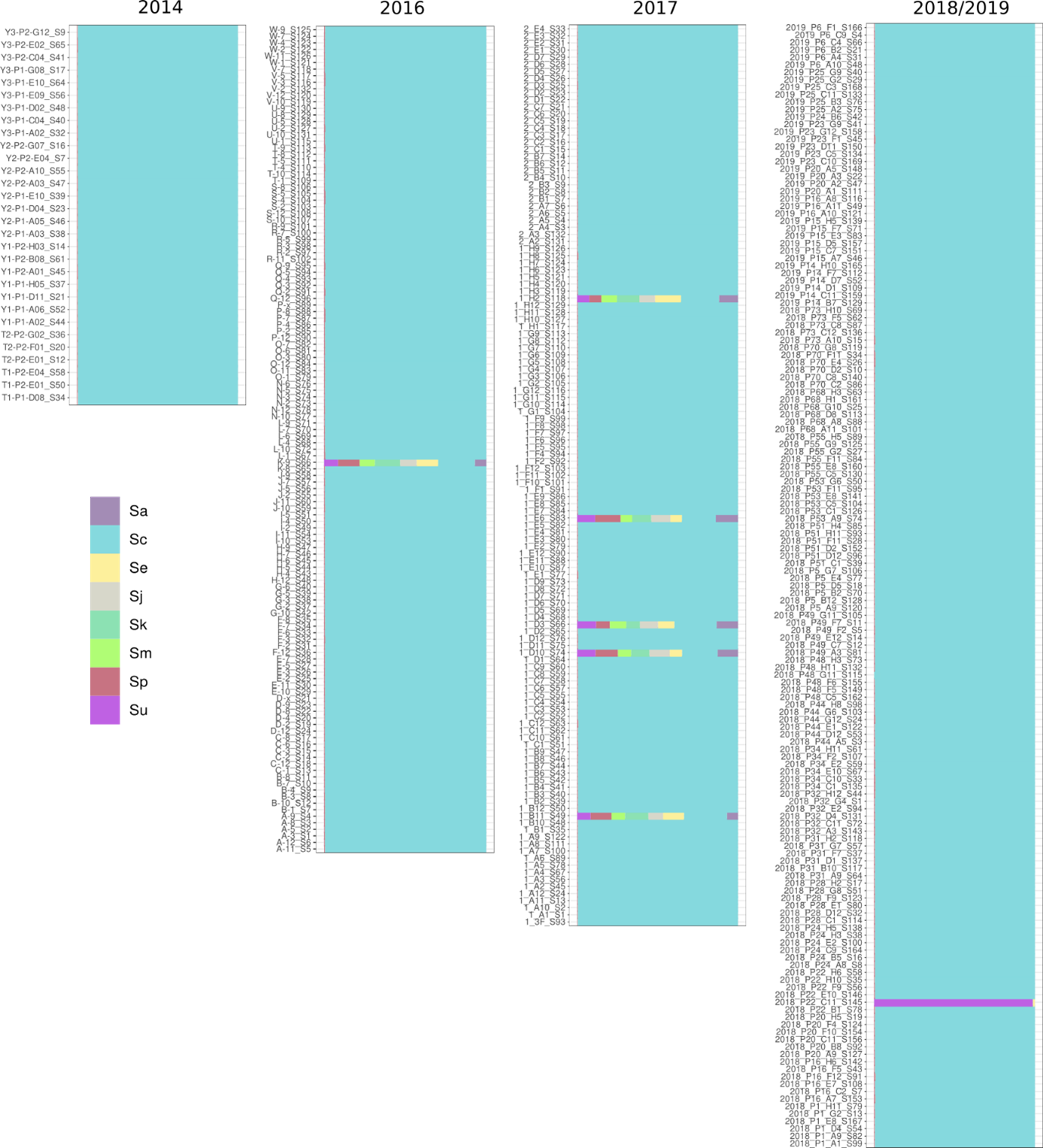

Supplement: S1 Fig — Proportion of 31-mers with an exact match to one of the sensu stricto Saccharomyces species S. arboricola (Sa), S. cerevisiae (Sc), S. eubayanus (Se), S. jurei (Sj), S. kudriavzevii (Sk), S. mikatae (Sm), S. paradoxus (Sp), and S. uvarum (Su) for each spontaneous isolate against a database of 31-mers unique to each species. (TIF) [file pgen.1011223.s001.tif]

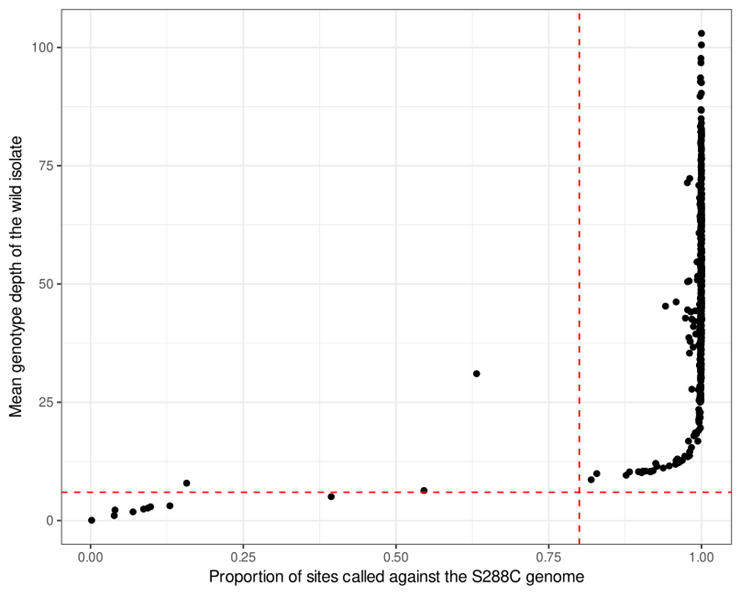

Supplement: S2 Fig — (TIF) [file pgen.1011223.s002.tif]

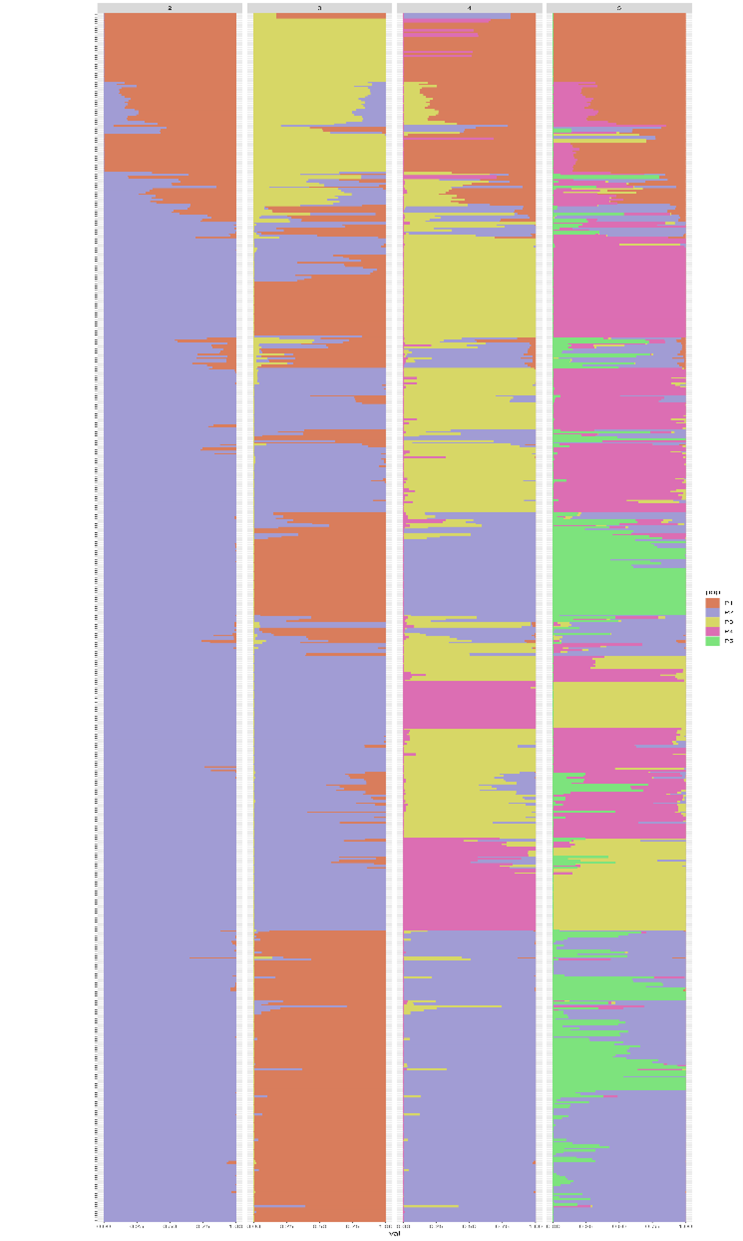

Supplement: S3 Fig — (TIF) [file pgen.1011223.s003.tif]

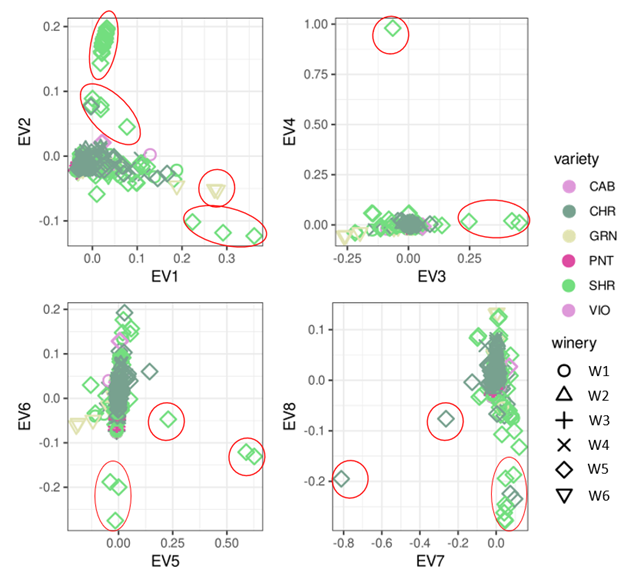

Supplement: S4 Fig — PCA outliers used to compare to spontaneous isolates with non-commercial wine ancestry are circled in red. (TIF) [file pgen.1011223.s004.tif]

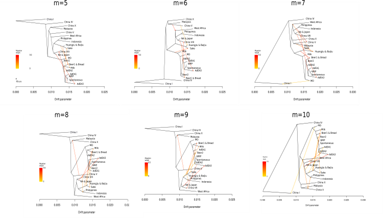

Supplement: S5 Fig — Migration edges are colored based on the estimated proportion of drift from source to target population. WRP represents the Wine Reference Panel. (TIF) [file pgen.1011223.s005.tif]

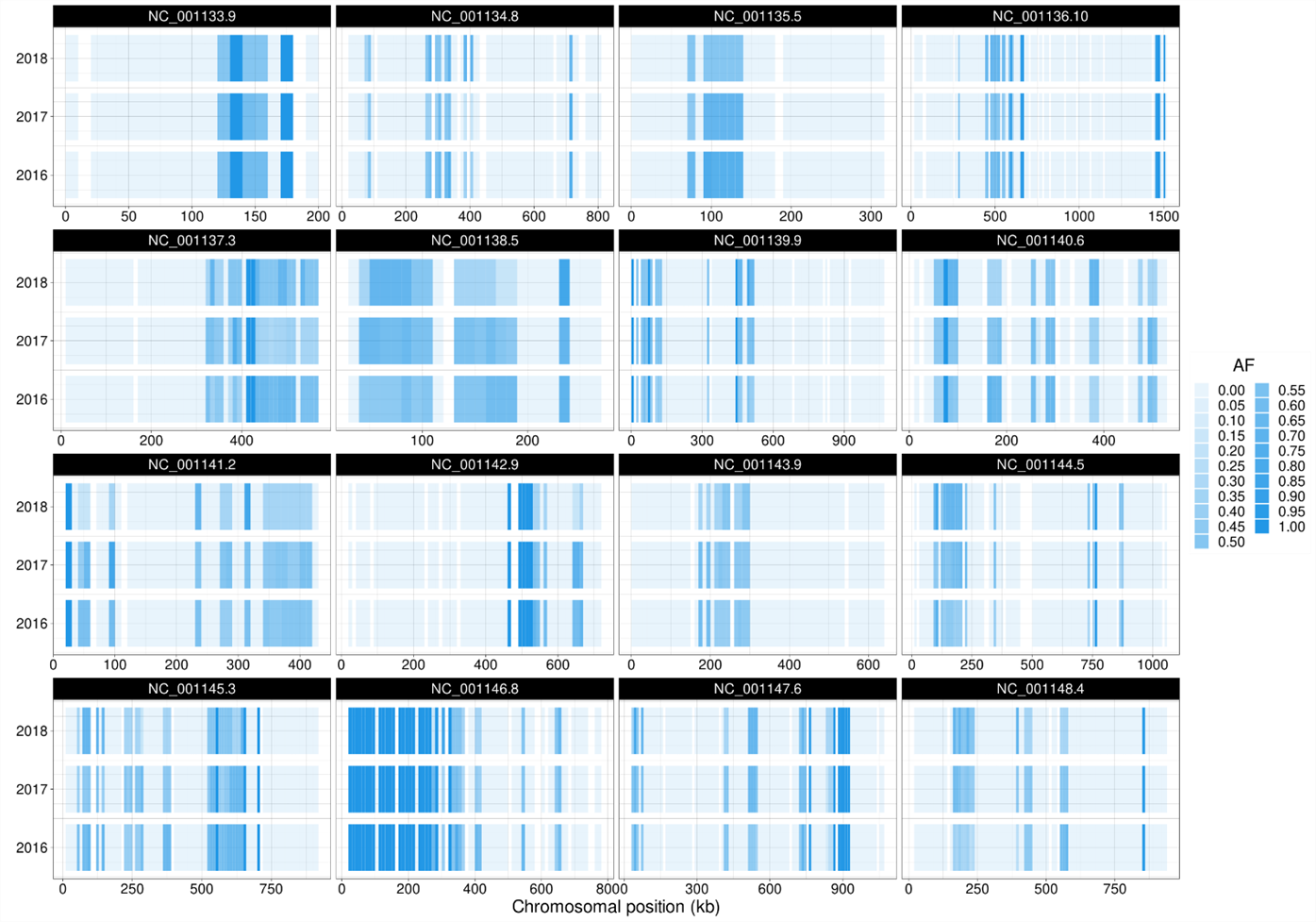

Supplement: S6 Fig — Blank windows are where the local topology was unresolved. (TIF) [file pgen.1011223.s006.tif]

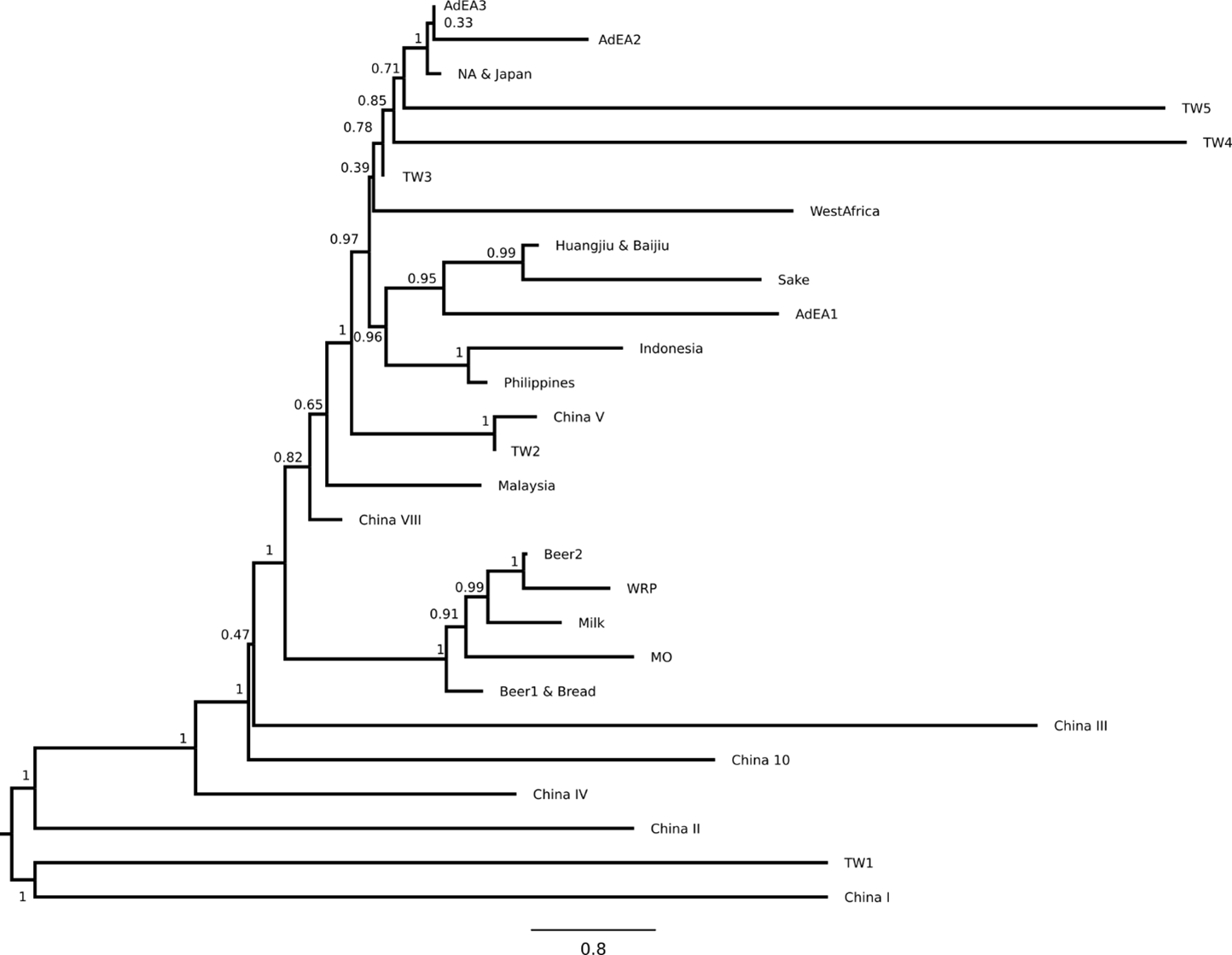

Supplement: S7 Fig — cerevisiae populations along with AdEA1, AdEA2 and AdEA3 using fixed admixture loci identified in Fig 3A. As no loci were fixed in AdEA2 a single homozygous diploid isolate from pop AdEA2 was used (Q-3_S92). Node posterior probability is shown. Scale is in coalescent units. (TIF) [file pgen.1011223.s007.tif]

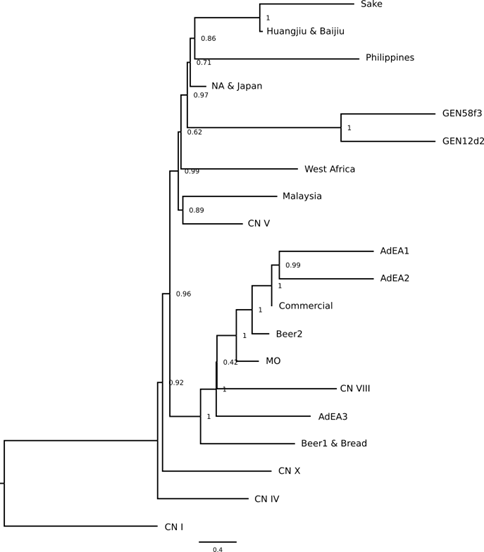

Supplement: S8 Fig — AdEA1, AdEA2 and AdEA3 are included as an indicator that although they contain admixture (~50% of the genome in AdEA3) they are still placed closer to domesticated lineages. Node posterior probability is shown. The scale is in coalescent units. (TIF) [file pgen.1011223.s008.tif]

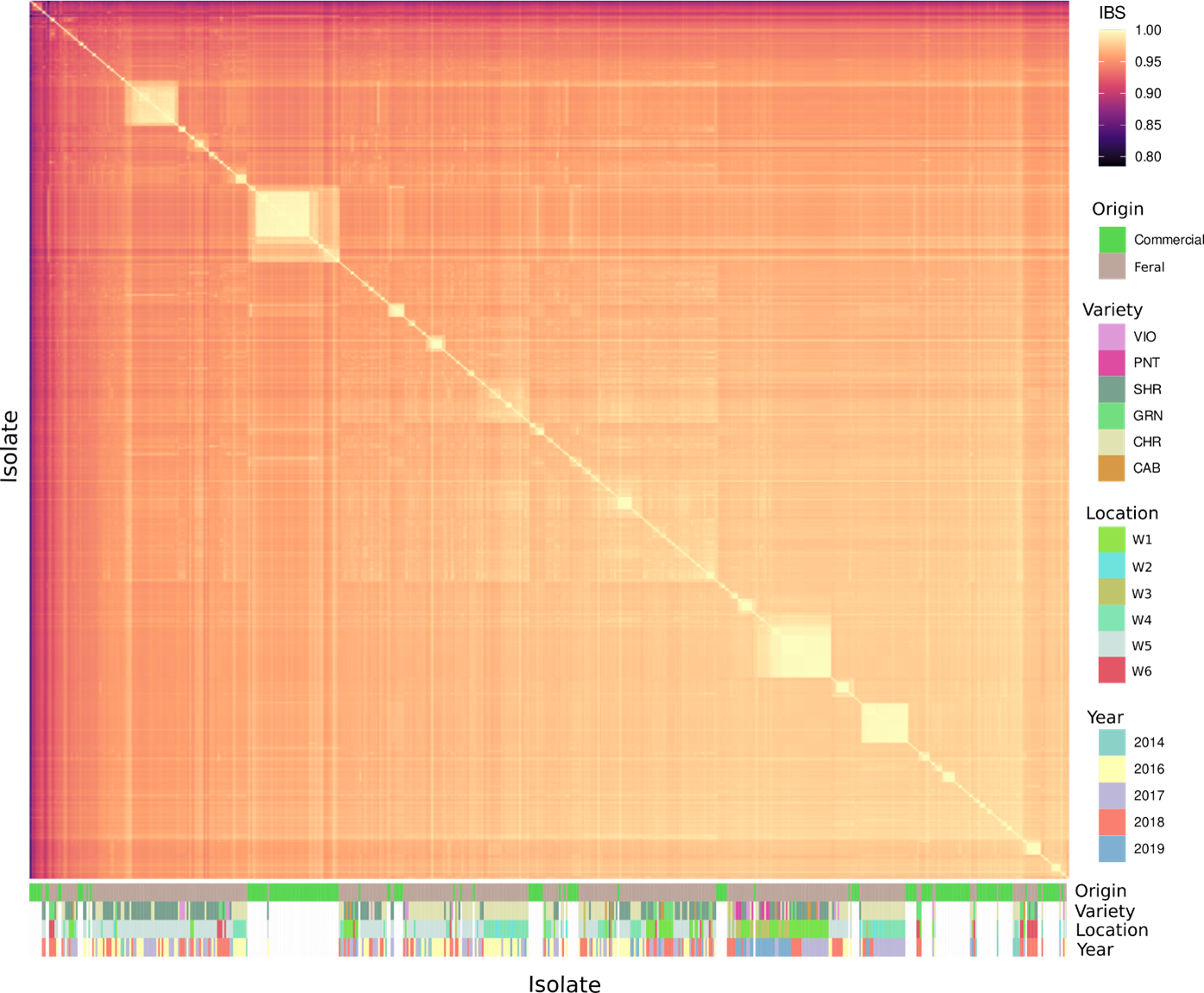

Supplement: S9 Fig — VIO = Viognier, PNT = Pinot Noir, SHR = Shiraz, GRN = Grenache, CHR = Chardonnay, CAB = Cabernet Sauvignon. (TIF) [file pgen.1011223.s009.tif]

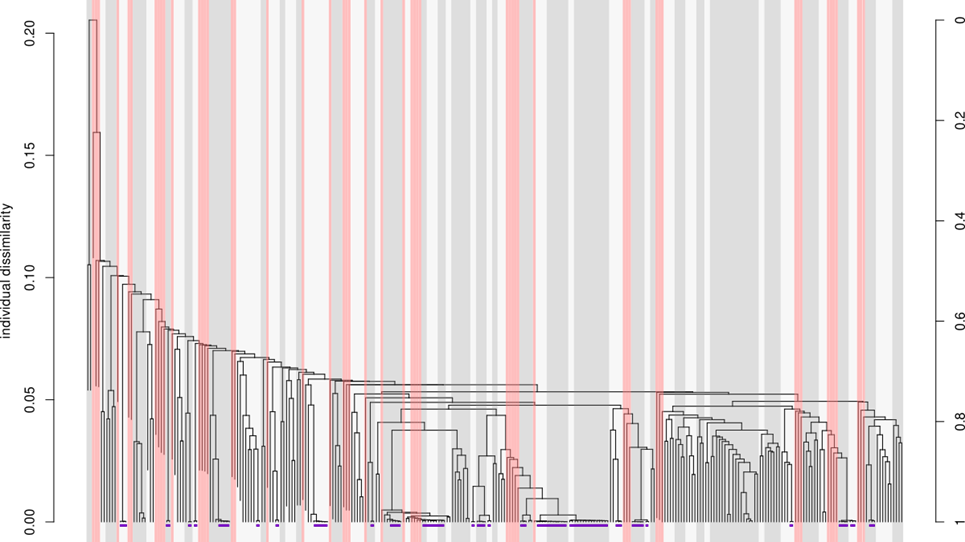

Supplement: S10 Fig — Alternation between grey and white represent lineages with individual isolates in their own lineage (outliers) highlighted in red. Purple bars represent individual isolates recognized as clonal clusters. A list of individuals and their lineage and clonal status can be found in S3 Table. (TIF) [file pgen.1011223.s010.tif]

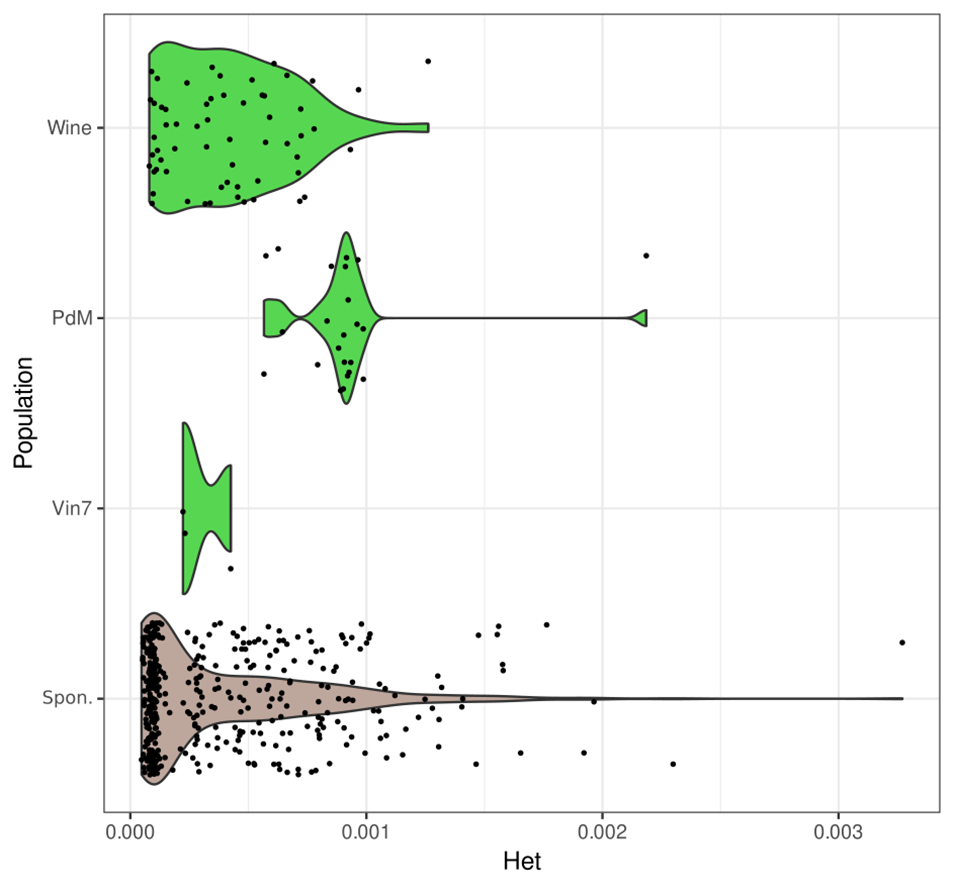

Supplement: S11 Fig — WRP isolates are broken down into their respective clades Wine = mixed European wine clade; PdM = Prise de mousse and Vin7. (TIF) [file pgen.1011223.s011.tif]

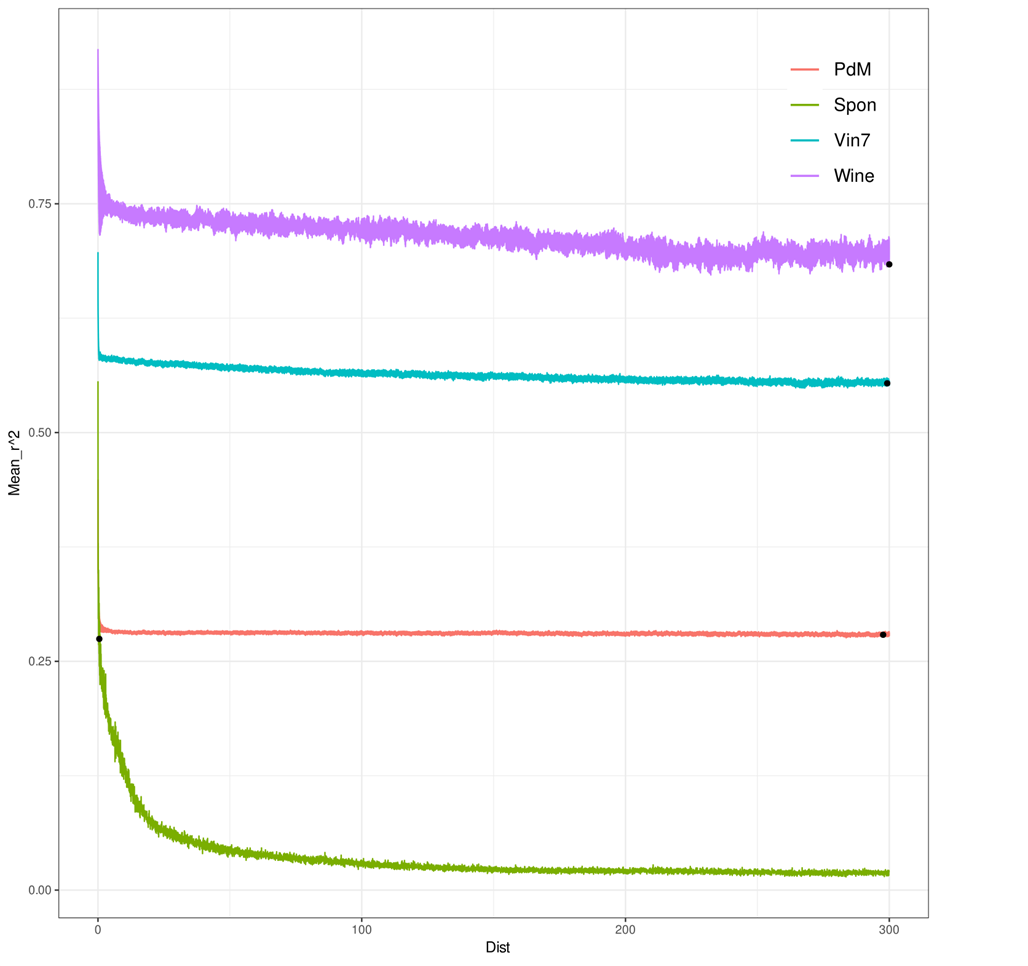

Supplement: S12 Fig — Distances where linkage disequilibrium decayed to half its total value are marked by a black point. (TIF) [file pgen.1011223.s012.tif]

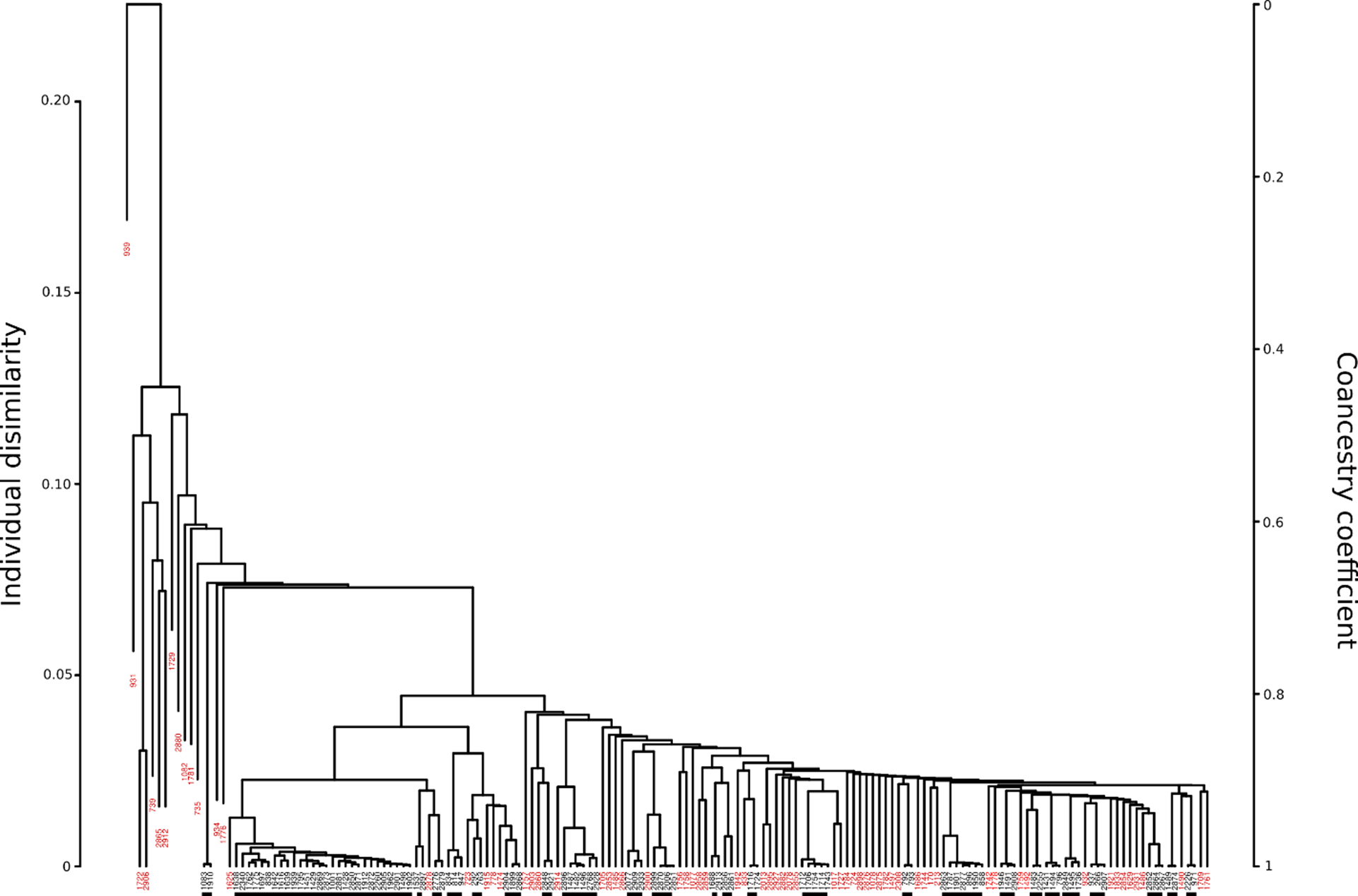

Supplement: S13 Fig — Red isolates are unique isolates that do not form a lineage. (TIF) [file pgen.1011223.s013.tif]

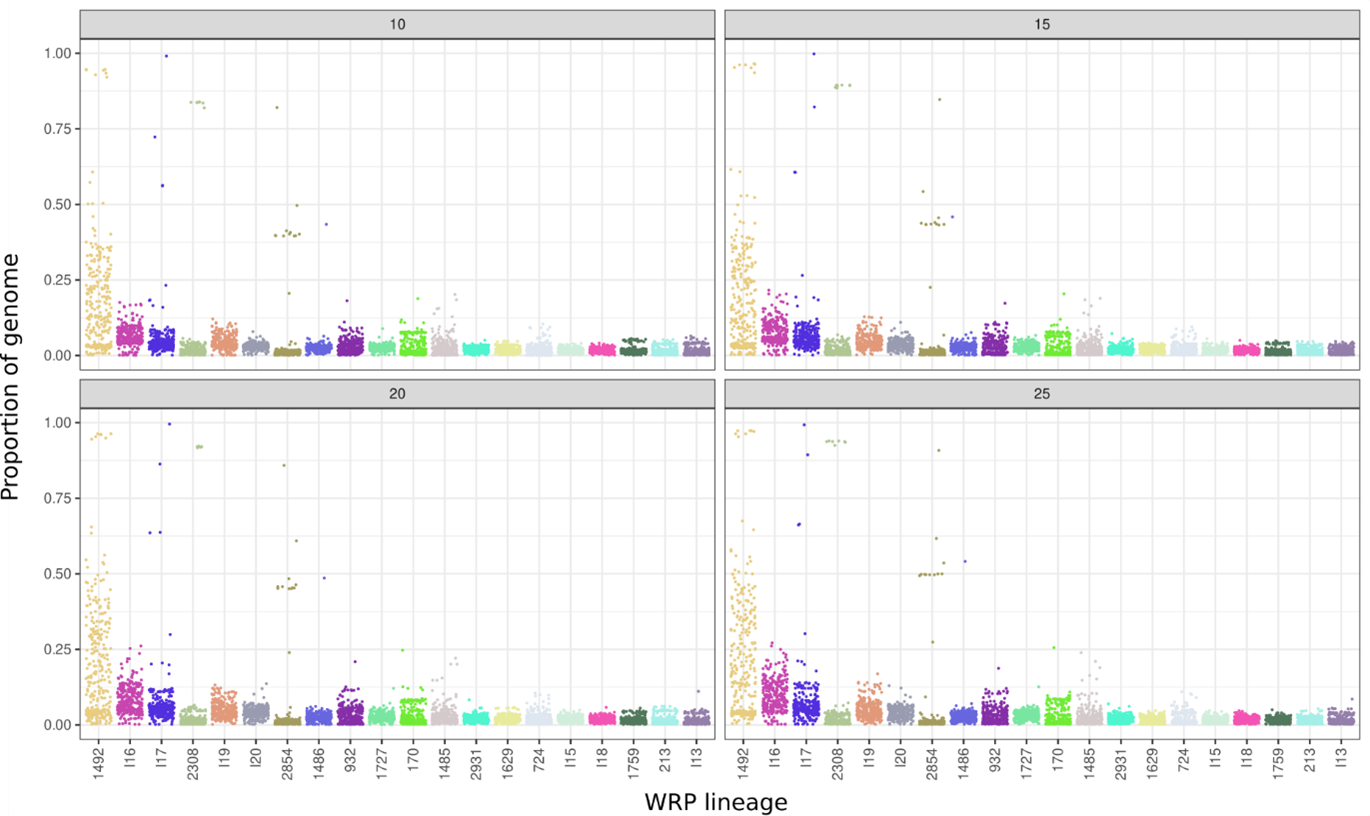

Supplement: S14 Fig — Four different window sizes 10, 15, 20 and 25kb were used to test for bias in window assignment based on window size. (TIF) [file pgen.1011223.s014.tif]

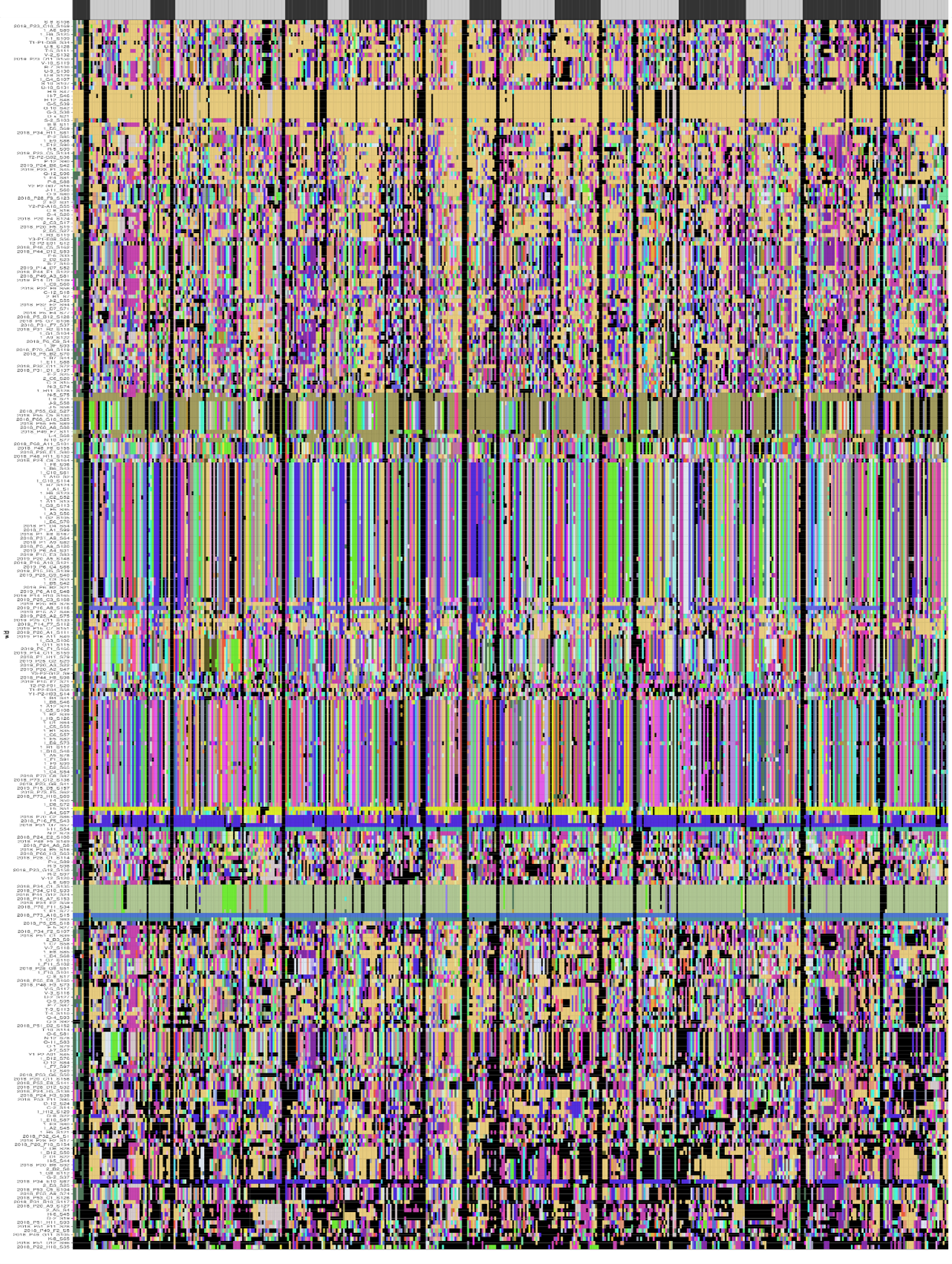

Supplement: S15 Fig — Colors indicate assigned WRP strain lineage of origin. Block-wise assignment to chromosomes can be found above the figure. (TIF) [file pgen.1011223.s015.tif]

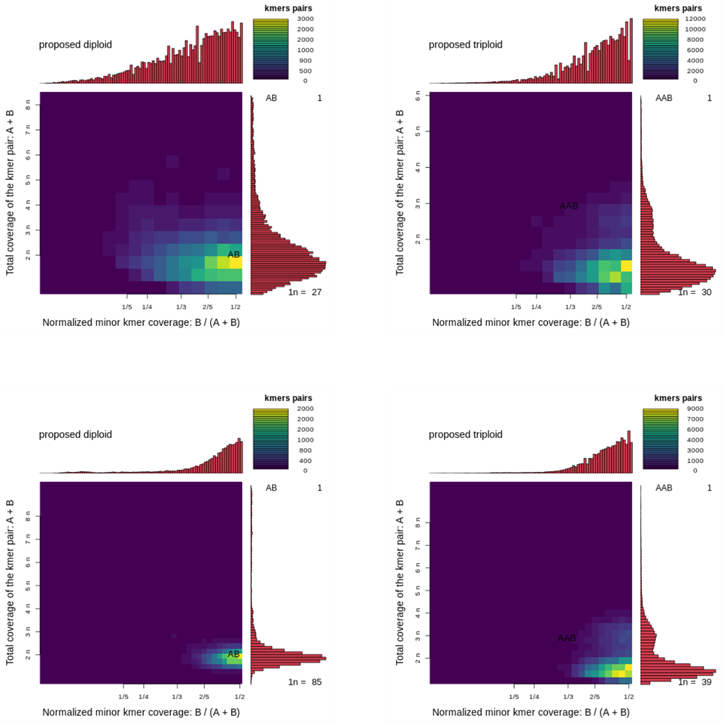

Supplement: S16 Fig — Upper: Spontaneous samples, Lower: WRP samples. (TIF) [file pgen.1011223.s016.tif]

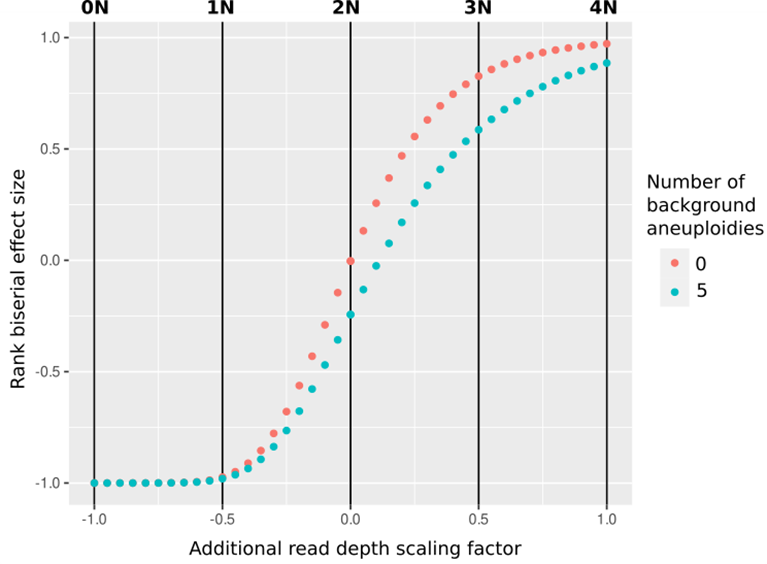

Supplement: S17 Fig — Aneuploidies were simulated in the genome wide background by scaling read depth for 0 and 5 chromosomes to 3N (ie, 1.5x chromosome read depth), aneuploid chromosomes were randomly selected from the empirically observed chromosome aneuploidy rate. Test chromosomes were scaled by a factor iterating through -1 to 1 (0N - 4N) by 0.1 to simulate read depth bias (2N read depth + 2N read depth x scaling factor). (TIF) [file pgen.1011223.s017.tif]

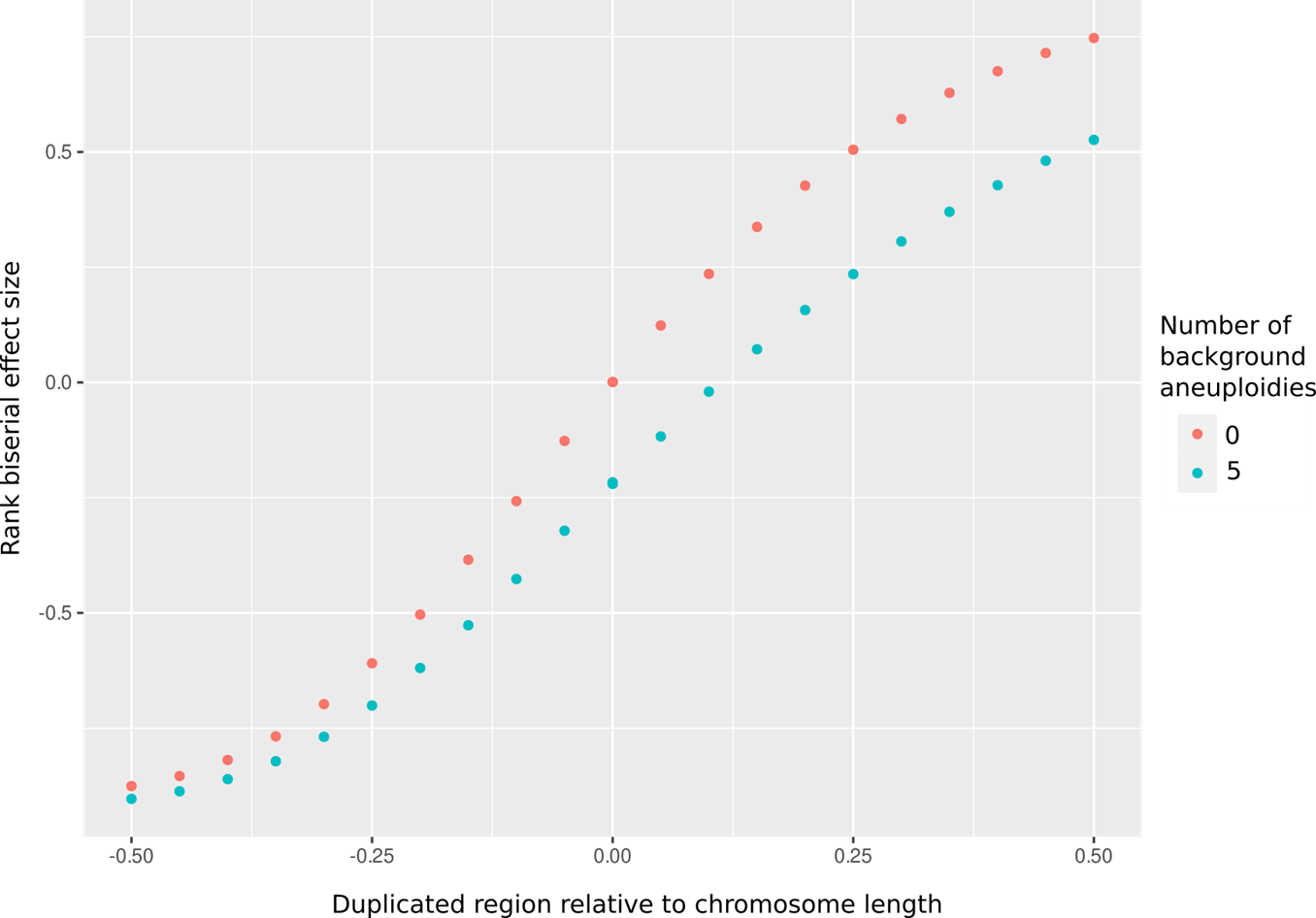

Supplement: S18 Fig — Duplications were simulated in the genome wide background by scaling read depth for 0 and 5 chromosomes to 3N (i.e., 1.5x chromosome read depth), aneuploid chromosomes were randomly selected from the empirically observed chromosome aneuploidy rate. Test chromosomes had a fraction of their length (-0.5–0.5) scaled by a factor of 1.5 (i.e. –0.5 has 1N for half the chromosome; 0.5 has 3N for half the chromosome). (TIF) [file pgen.1011223.s018.tif]

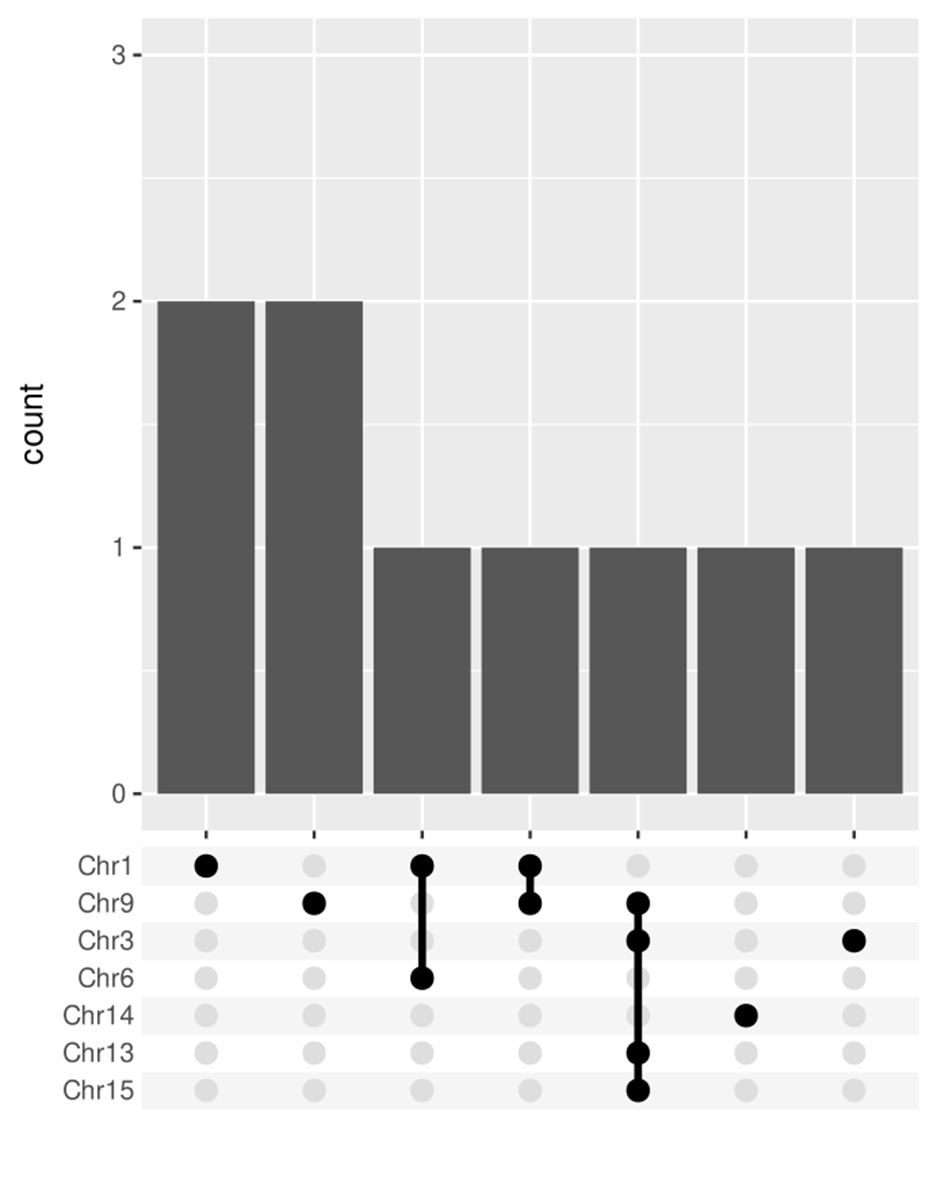

Supplement: S19 Fig — Upset plot nodes represent aneuploidies that were present simultaneously in the same individual. (TIF) [file pgen.1011223.s019.tif]

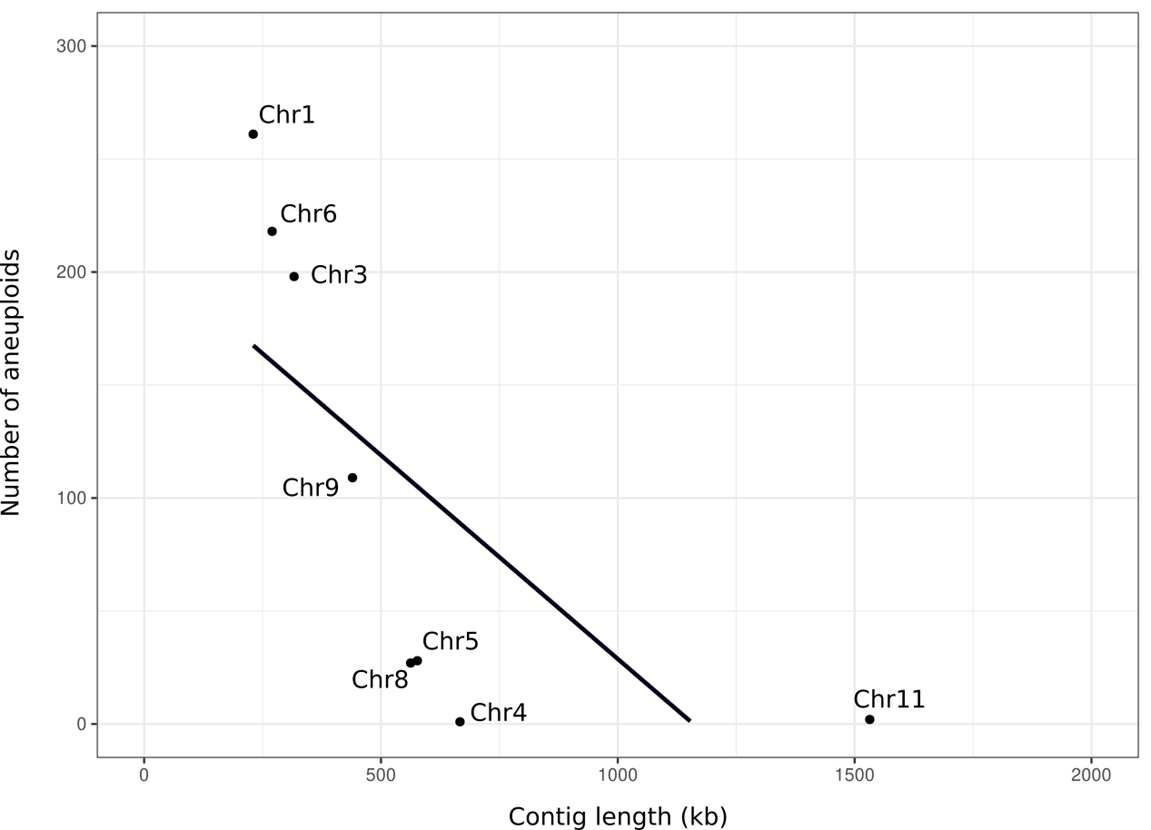

Supplement: S20 Fig — Linear model fit to the number of observations of aneuploidies for each chromosome across the entire spontaneous isolate pool against chromosome size. (TIF) [file pgen.1011223.s020.tif]

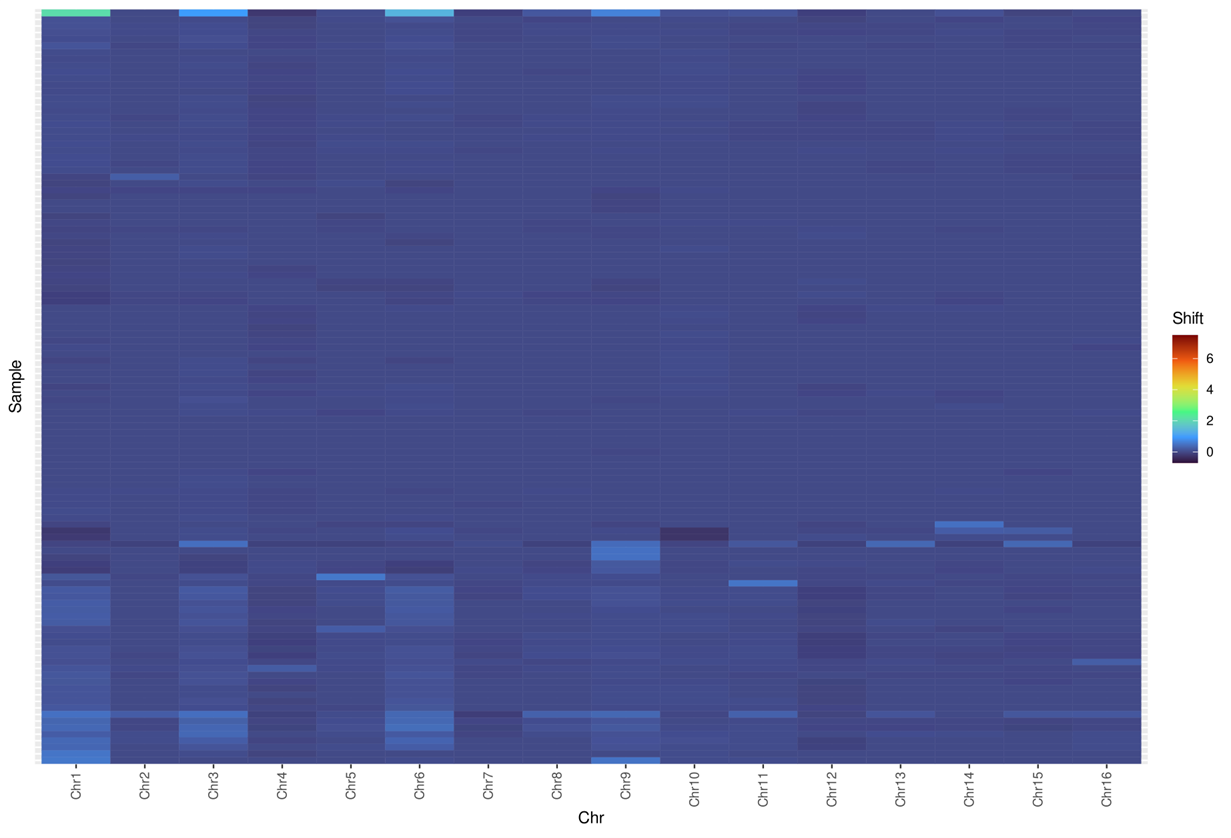

Supplement: S21 Fig — Shift values represent estimated location shift in read depth distribution across each chromosome divided by the median background read depth. Therefore, location shift values represent gain or loss of chromosomal read depth against the background distribution, i.e. 0.5 shift per chromosome duplication. Figure legend is scaled to be more easily compared to Fig 5B. (TIF) [file pgen.1011223.s021.tif]

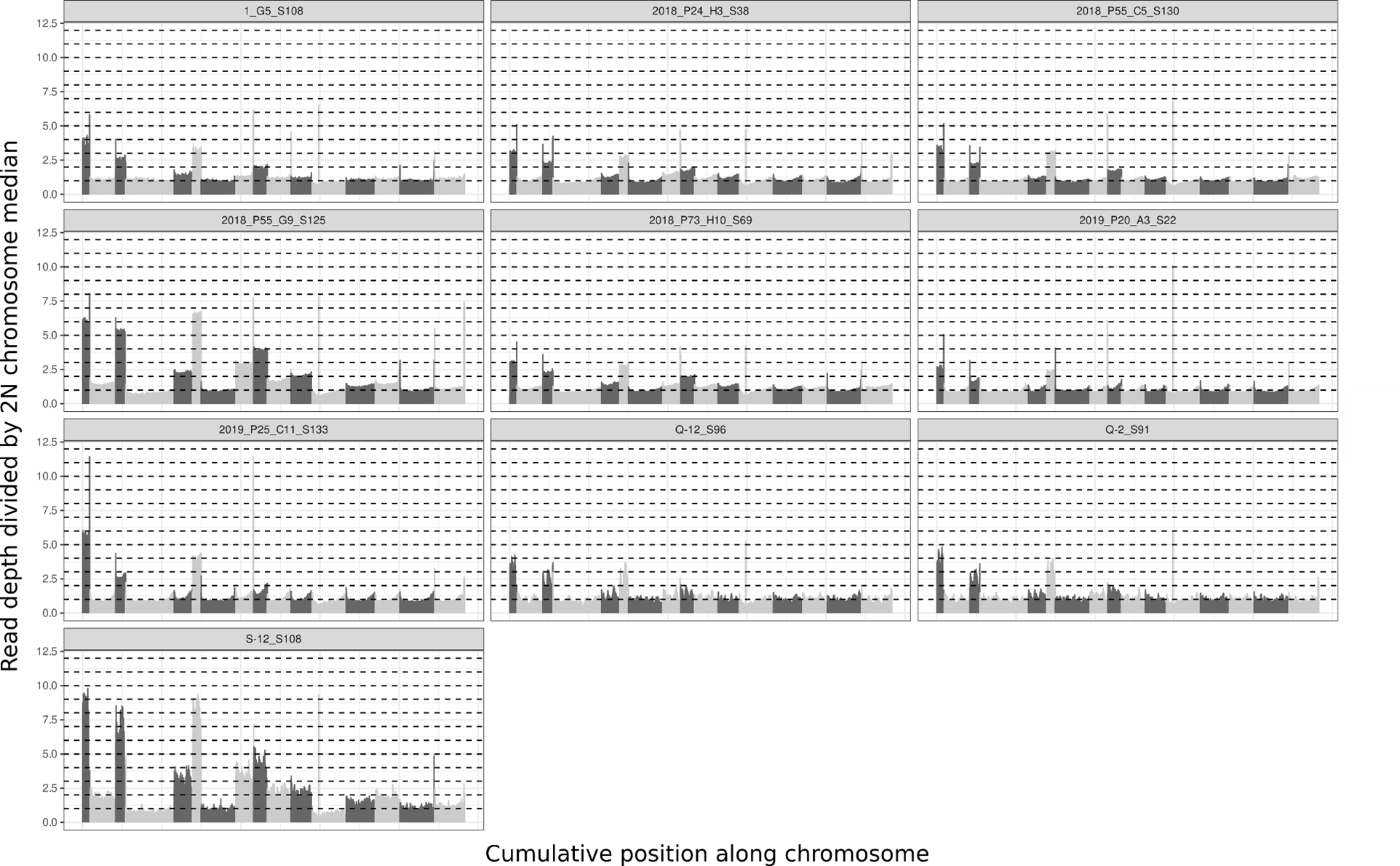

Supplement: S22 Fig — Dashed horizontal lines denote expected copy number driven read depth increases (0.5 per 1N gain). (TIF) [file pgen.1011223.s022.tif]

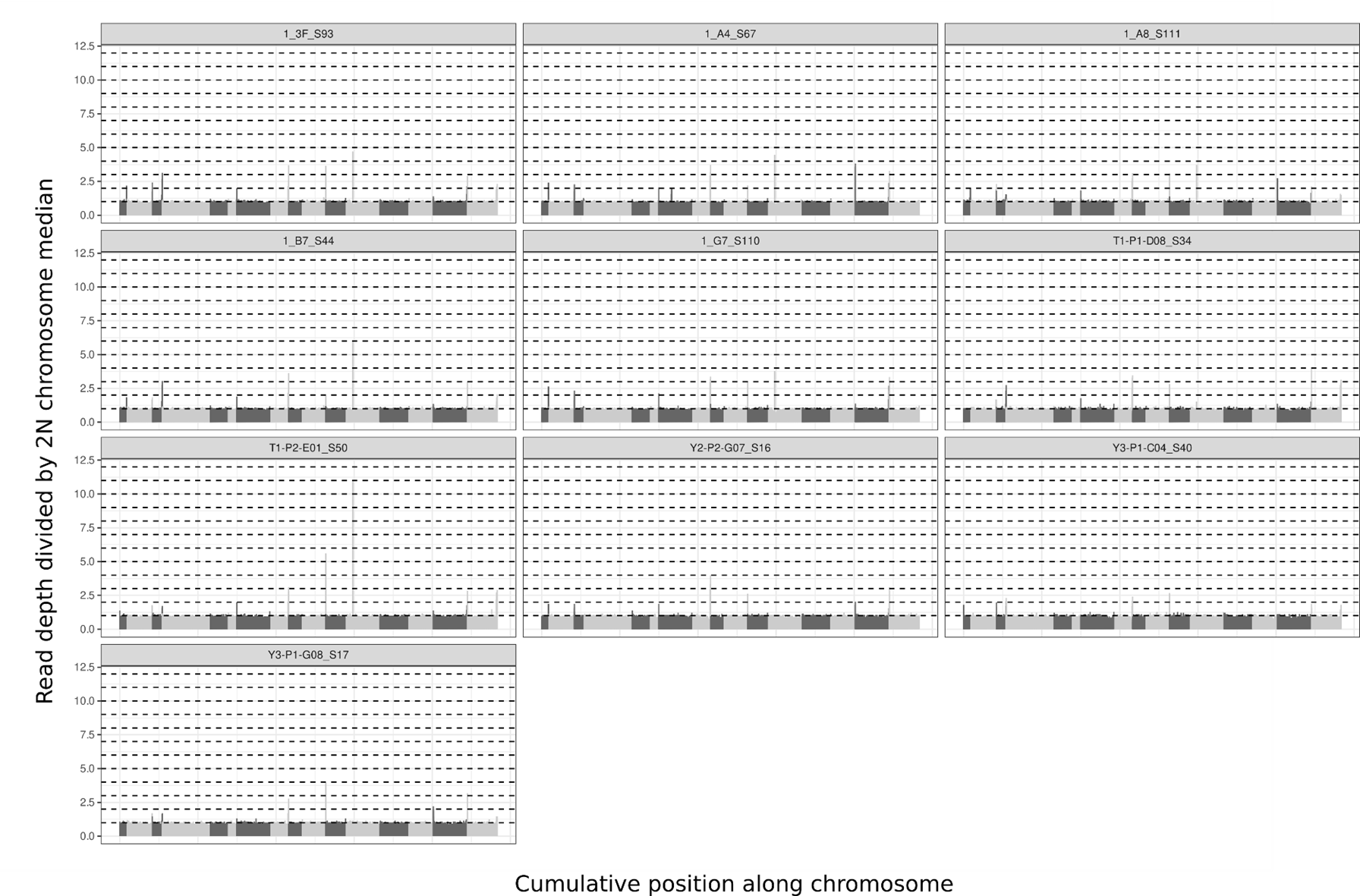

Supplement: S23 Fig — Dashed horizontal lines denote expected copy number driven read depth increases (0.5 per 1N gain). The y axis is scaled to match S22 Fig. (TIF) [file pgen.1011223.s023.tif]

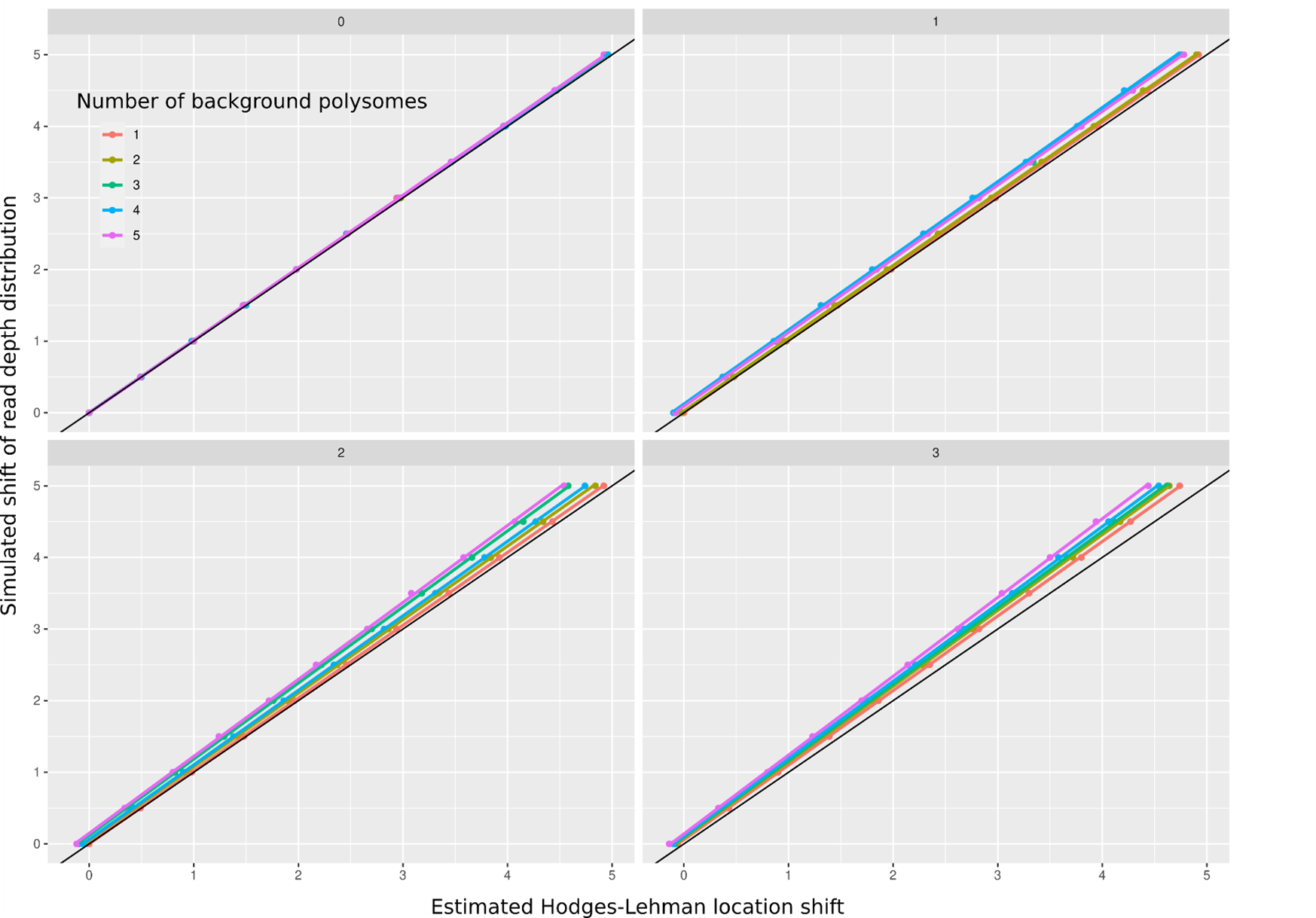

Supplement: S24 Fig — Hodges-Lehman location shift in simulated polysomes for polysomes shifted by 0–5 by 0.5 (where 0.5 corresponds to a 1N increase in polysome copy number) against genome wide read depth data that has simulated 0–5 background polysomes co-occurring with the test chromosome. Simulated background polysomes had their read depth shifted by a factor of 0–3 to simulate 2N-8N copy number for background polysomes. (TIF) [file pgen.1011223.s024.tif]
